# A Global Systematic Review on the Potential of Metal-Based Nanoparticles in the Fight Against Mosquito Vectors

**DOI:** 10.1155/jotm/2420073

**Published:** 2025-06-09

**Authors:** Awoke Minwuyelet, Delenasaw Yewhalaw, Yibeltal Aschale, Andrea Sciarretta, Getnet Atenafu

**Affiliations:** ^1^Department of Biology, College of Natural and Computational Science, Debre Markos University, Debre Markos, Ethiopia; ^2^Tropical and Infectious Diseases Research Center, Jimma University, Jimma, Ethiopia; ^3^School of Medical Laboratory Sciences, Faculty of Health Sciences, Jimma University, Jimma, Ethiopia; ^4^Department of Medical Laboratory Science, College of Health Sciences, Debre Markos University, Debre Markos, Ethiopia; ^5^Department of Agricultural, Environmental and Food Sciences, University of Molise, Campobasso, Italy

**Keywords:** *Anopheles*, biosynthesized, *Culex aedes*, larvicidal activity, metal-based nanoparticles, mosquitocidal effect, vector-borne disease

## Abstract

**Background:** Mosquito-borne diseases, such as malaria, filariasis, dengue, chikungunya, Zika, and other viral infections, pose significant public health challenges worldwide. For many years, chemical insecticides were used in the form of indoor residual spraying (IRS) and insecticide-treated nets (ITNs). However, these methods have encountered several limitations such as the development of resistance, environmental impact, and nontarget effects. In recent years, metal-based nanoparticles (MNPs) have emerged as a promising alternative in the fight against mosquito vectors. This systematic review aimed to explore the potential application of MNPs in combating medically significant vectors.

**Methods:** Global databases such as PubMed, Scopus, Web of Science, and ProQuest were used to search for relevant articles published from 2011 to 2021. The data search was conducted between July 30 and August 15, 2022. Keywords such as “Metal-based nanoparticles,” “Nanoparticles toxicity,” “Mosquito control,” “Larvicidal,” “Nanomaterials in mosquito control,” and “biosynthesized” were used both individually and in combination to find pertinent studies. Only original articles published in English that offered comprehensive information on the effects of biosynthesized MNPs on mosquitoes were included in the study. These articles were selected based on the presence of key details such as the type and source of nanoparticles (NPs), size range (1–100 nm), and the mosquito larval species tested, exposure duration, and corresponding lethal concentration (LC) levels. Studies lacking sufficient data or with unavailable full texts were excluded from the analysis. The quality of each original article was evaluated using a standardized quality assessment tool adapted from the Joanna Briggs Institute (JBI) Critical Appraisal Checklist. Data were extracted from texts, tables, and figures of the included articles, and their validity was assessed using standardized tools.

**Result:** A total of 65 articles were included, covering laboratory and field findings on NPs such as silver (Ag), gold (Au), palladium (Pd), cobalt (Co), titanium dioxide (TiO_2_), nickel (Ni), copper (Cu), cadmium (Cd), selenium (Se), zinc (Zn), magnesium oxide (MgO), iron and iron oxide (Fe and Fe_2_O_3_), and aluminum oxide (Al_2_O_3_). Among these, AgNPs and CuNPs were the most extensively tested and found effective against various larval instars, pupae, and adults of *Aedes aegypti*, *Ae*. *albopictus*, *Anopheles stephensi*, *An*. *subpictus*, *Culex quinquefasciatus*, and *Cx*. *pipiens*, with satisfactory 50% and 90% LC values.

**Conclusions:** The study highlighted the promising potential of MNPs as effective agents for controlling mosquito vectors, particularly at various developmental stages of *Anopheles*, *Culex*, and *Aedes* species. Most studies focused on AgNPs and AuNPs, with some attention given to other MNPs. Notably, NPs synthesized from plant extracts such as *Azadirachta indica* and microorganisms demonstrated strong larvicidal activity, especially against *Culex*, *Anopheles*, and *Aedes* larvae. Efficacy varied across developmental stages, with first-, third-, and fourth-instar larvae being most susceptible. These findings underscore the potential of MNPs as an environmentally friendly alternative to conventional mosquito control methods.

**Implications of Key Findings:** MNPs, especially Ag and AuNPs, are effective larvicides targeting the early developmental stages of mosquitoes. These NPs, derived from plants and microorganisms, demonstrate an environmentally friendly, cost-effective insecticidal effect and could serve as alternatives to chemical insecticides. However, further research is needed to optimize their synthesis, application, and scalability for large-scale use. Additionally, the varying efficacy of different mosquito species and life stages requires a more targeted, species-specific use of NPs, along with ongoing environmental assessments to ensure their long-term safety and effectiveness.

## 1. Introduction

Despite their small size, mosquitoes have a profound impact on global health due to their ability to transmit a wide range of diseases. This makes them one of the most known and targeted groups in public health sectors. With over 120 genera within the family Culicidae and the order Diptera, understanding the diversity of mosquitoes is crucial to recognizing their various roles in disease transmission across different regions [1, 2]. Among these, some mosquito genera play significant roles in the transmission of various infectious diseases: *Anopheles* is the primary vector for malaria and lymphatic filariasis (LF). *Aedes* species transmit viral diseases such as dengue, Zika, chikungunya, and yellow fever, as well as LF. *Culex* mosquitoes are responsible for spreading viral infections like West Nile virus infection and encephalitis, and also for parasitic diseases such as LF. *Mansonia* mosquitoes primarily transmit LF. *Ochlerotatus* (a subgenus of *Aedes*) spreads Eastern equine encephalitis (EEE), while *Coquillettidia* mosquitoes are associated with certain encephalitis virus infections. Together, these mosquito genera serve as crucial vectors for a variety of infectious diseases that affect millions of people worldwide [3–5].

The most notable mosquito-borne diseases include malaria, dengue fever, yellow fever, filariasis, encephalitis, Zika, and West Nile virus infection, along with other viral infections such as Sindbis and Usutu virus infections. These parasites and viruses, which affect millions globally, are primarily transmitted through the bites of infected female mosquitoes from the *Anopheles*, *Culex*, and *Aedes* genera [6–10]. The re-emergence of these diseases in recent years can be attributed to factors like global climate change, rapid urbanization, increased international travel, and the expansion of mosquito populations [2, 11–15].

Malaria is a life-threatening infectious disease caused by protozoan parasites of the genus *Plasmodium*. It is one of the most significant mosquito-borne diseases and is transmitted through the bites of infected female *Anopheles* mosquitoes [14–16]. According to the World Health Organization's (WHO) 2023 malaria report, an estimated 249 million malaria cases and 608,000 deaths occurred worldwide in 2022. Sub-Saharan Africa bears the brunt of this burden, accounting for 94% of cases and 95% of deaths. Children under five and pregnant women are particularly vulnerable to malaria. For example, in 2022, 80% of malaria deaths in Africa were among children under five [17]. Although there are over 500 species of *Anopheles* mosquitoes globally, the predominant species responsible for malaria transmission worldwide include the *An*. *gambiae* complex, *An*. *funestus*, *An*. *stephensi*, *An*. *dirus*, *An*. *albimanus*, *An*. *minimus*, and *An*. *arabiensis* [18–24]. Similarly, other diseases transmitted by the bite of infected *Anopheles* species include the following: LF, which is caused by filarial worms (*Wuchereria bancrofti*, *Brugia malayi*, and *Brugia timori*). LF can lead to severe disability and elephantiasis [10, 25]. Globally, over 120 million people are infected, with 40 million disfigured or incapacitated by the disease [10, 17, 25, 26]. LF is primarily transmitted by mosquito vectors, with *Anopheles* species being a major contributor, particularly in West Africa [27]. *Anopheles* species that transmit LF include *An*. *gambiae* and *An*. *funestus* in Africa. Additionally, LF is also transmitted by certain species of *Culex* and *Mansonia* mosquitoes in urban and semiurban areas, while *Anopheles* are mainly found in rural regions and *Aedes* primarily in endemic islands in the Pacific [26, 28–34].

In addition to diseases transmitted by Anopheles mosquitoes, such as malaria, LF several other significant illnesses are primarily spread by Aedes mosquitoes. Notably, dengue fever, yellow fever, chikungunya, and Zika are among the most prevalent. These diseases pose substantial public health challenges in tropical and subtropical regions worldwide [10, 25, 35–37]. Dengue fever is caused by different strains of dengue virus (Flaviviridae, *Flavivirus*) serotypes (DENV-1, DENV-2, DENV-3, and DENV-4) [38]. Dengue causes a broad spectrum of clinical manifestations, ranging from asymptomatic or mild illness to more severe flu-like symptoms, which can include high fever, severe headache, pain behind the eyes, joint and muscle pain, rash, and nausea. In some cases, it can progress to more severe forms, such as dengue hemorrhagic fever or dengue shock syndrome [10]. It is the most common disease transmitted by *Aedes aegypti and Ae*. *albopictus* that affect over 80 countries and five WHO regions: Africa, Americas, Southeast Asia, Western Pacific, and Eastern Mediterranean Regions globally [10, 36, 39]. In 2023, the highest number of dengue cases was recorded, impacting over 80 countries across all WHO regions. Since the start of the year, continuous transmission and an unexpected surge in cases have led to an unprecedented total of over 6.5 million dengue cases and more than 7300 dengue-related deaths [40].

Yellow fever (Flaviviridae, *Flavivirus*) is a viral hemorrhagic disease primarily transmitted through the bite of infected female *Ae*. *aegypti* mosquitoes, though *Ae*. *albopictus* and *Haemagogus* species can also serve as vectors [36, 37, 41]. It is characterized by fever, jaundice, and in severe cases, organ failure, and can be fatal without proper treatment. Yellow fever remains a significant public health threat, especially in tropical and subtropical regions of Africa and the Americas [41–45].

Similarly, Zika is a disease caused by the Zika virus (Flaviviridae, *Flavivirus*) which is a cause of neurological disorders/neonatal malformations such as Guillain-Barré syndrome and fetal microcephaly [8]. It is primarily transmitted by the bite of infected female *Ae*. *aegypti* and occasionally by *Ae*. *albopictus* and has emerged as a major public health concern [10, 35, 46–48]. Although its incidence has generally been low from 2018 to the present, Zika continues to pose a risk in many tropical and subtropical regions [45, 48, 49].

Chikungunya is caused by a single-stranded positive-sense RNA virus and is introduced to humans through the bite of infected *Ae*. *aegypti* and *Ae*. *albopictus* mosquitoes [35, 48, 50, 51]. The spread of the Chikungunya virus is a significant public health concern in many countries, including America. In 2023, there were over 120,000 cases and 51 deaths, with 46 of these fatalities occurring in Paraguay [35, 52].

Likewise, among the diseases transmitted by *Culex* species mosquitoes, notable examples include West Nile virus disease, which is caused by a single-stranded, positive-sense RNA virus (the genus *Flavivirus*) and primarily transmitted by particularly biting of infected female *Cx*. *pipiens* and *Cx*. *quinquefasciatus*. The disease is endemic in parts of Africa, Europe, Asia, and North America, with periodic outbreaks causing significant public health impacts [53]. Similarly, Japanese encephalitis is transmitted by *C*x. *tritaeniorhynchus* in Asia and *Cx*. *vishnui* in some parts of Southeast Asia. It is a leading cause of viral encephalitis in Asia and Australia, with high mortality rates and long-term neurological sequelae among survivors. It affects rural and agricultural areas, particularly during the rainy season, and poses a substantial burden on affected communities [54, 55].

While human infections with the Sindbis virus (Togaviridae, Alphavirus) typically result in symptoms such as febrile illness, arthritis, and a maculopapular rash, these symptoms can vary in severity; some individuals may experience joint pain and inflammation. Recent studies have detected the Sindbis virus in *Culex* mosquitoes, suggesting that it is primarily transmitted by *Cx*. *pipiens*, *Cx*. *torrentium*, and *Cx*. *perexiguus* [56–59]. Finally, the Usutu virus (Flaviviridae, *Flavivirus*) is an emerging virus that belongs to the Japanese encephalitis virus (JEV) complex. Although the Usutu virus was responsible for significant outbreaks in blackbirds (*Turdus merula*) and gray owls (*Strix nebulosa*), it has also been detected in *Culex* mosquitoes, which may serve as potential vectors for the virus [60–62]. To prevent mosquito-borne diseases, chemical insecticides have been extensively used since the 1940s [6, 63]. Despite their effectiveness in reducing vector-borne diseases, mosquitoes' diverse breeding habitats, resting sites, and insecticide resistance behaviors mean these diseases remain significant public health issues [64, 65]. Additionally, the persistent use of insecticides has led to ecological imbalances and bioaccumulation of substances in soil, food, water, and the environment, causing harmful effects on nontarget organisms and humans [65, 66].

Even with these drawbacks, the WHO continues to recommend chemical insecticides as the primary method for vector control, including their use in insecticide-treated nets (ITNs), IRS, open spraying for adults mosquito control, and larvicides [67, 68]. Complementary methods like environmental management, protective clothing, and repellents are also employed [69, 70]. However, due to the severe environmental and health impacts, integrated vector management (IVM) strategies, where chemicals are one of several control methods, offer a more sustainable approach.

Integrated vector control strategies are currently the most effective for reducing mosquito populations [67]. These measures include the use of insecticides, environmental management, and biological control methods based on understanding mosquito biology, ecology, and pathogen interactions [71]. Biological control, using natural enemies to eliminate vectors, is a sustainable and environmentally friendly alternative unlikely to cause resistance [72, 73]. It is derived from botanical and microbial sources, and biological control methods are promising compared to chemical insecticides [71, 74–82].

Various MNPs control vector-borne diseases effectively through their unique properties. For example, MNPs such as silver, copper, and zinc oxide possess antimicrobial and pesticidal properties, disrupting mosquito biology by inducing oxidative stress, inhibiting reproduction, and cell damage that used to control vector-borne diseases [83, 84]. Their small size and large surface area allow for increased reactivity and interaction with mosquito bodies, increasing their toxic effect or repellent properties. Additionally, MNPs exhibit varying toxicity profiles for different mosquito species, reducing the risk of resistance development compared to traditional insecticides. Furthermore, when used in controlled concentrations, they are environmentally safe, with minimal toxicity to nontarget organisms. These combined benefits make MNPs a promising and sustainable alternative to conventional vector control methods [83, 85, 86]. These particles could be synthesized from plants and microorganisms [84, 86–88].

Plant parts (roots, leaves, flowers, fruits, peels, and stems) and microorganisms (fungi and bacteria) are rich in active ingredients that are used in various therapeutic industries, including biomedical, healthcare, and agriculture [89–100]. Nanoparticles can be synthesized using advanced technology from plant extracts using universal solvents and from microorganisms using various enzymes. These solvents and enzymes act as reducing agents, transforming heavy metal salts into MNPs [101, 102]. By regulating synthesis factors such as pH, temperature, incubation period, and mixing ratio, stable and uniformly sized NPs can be produced. Biologically synthesized NPs have been found to be more pharmacologically active than chemically synthesized ones [102].

Nanoparticles are microscopic particles with at least one dimension, typically less than 100 nanometers (nm), and exhibit unique physical and chemical properties due to their small size and high surface area-to-volume ratio. These properties include enhanced strength, catalytic activity, optical features (such as fluorescence), and magnetic behavior, which vary based on their composition and size [101, 103, 104]. Nanoparticles have gained significant interest for their potential applications in various sectors, including agriculture and biomedicine [101, 102]. These NPs have shown significant applications in medicine, including pathogen detection, drug delivery, and inhibition of insect growth [105, 106]. However, the small size and unique properties of NPs raise concerns about their potential environmental and health impacts.

Interestingly, MNPs can be engineered for targeted delivery and controlled release, minimizing environmental impact and improving specificity. Recent studies have demonstrated the effectiveness of MNPs against different mosquito species at various life stages, indicating their potential as a versatile tool in IVM strategies. For example, recent research indicates that NPs are safe for humans and the environment and are cost-effective for mosquito control [73, 107, 108]. Metal nanoparticles derived from natural sources, including gold, silver, palladium, platinum, zinc, and others, have garnered attention due to their eco-friendly applications [105, 109, 110]. These nanoparticles exhibit distinct physicochemical properties that enhance their effectiveness as insecticidal agents [73, 108, 111–113].

Different NPs derived from plant parts, microorganisms, and other chemicals have demonstrated the potential to disrupt various biological processes in mosquitoes, including growth, reproduction, and cellular function [73, 108, 111, 113]. For example, a study by Naveenkumar et al. (2023) revealed that silver nanoparticles (AgNPs) synthesis from *Gracilaria corticata* red seaweed approved larvicidal behavior on the fourth-instar larvae of *Ae*. *aegypti*, *An*. *stephensi*, and *Cx*. *quinquefasciatus* [108]. Similarly, other studies revealed that AgNPs have severe degradation in the larvae's hindgut, epithelial cells, midgut, and cortical area by inhibiting the acetylcholine esterase, *α*- and β-carboxylesterases, and phosphatase activities of 4^th^-stage larvae [111–113] and induced a redox imbalance in the larvae, marked by an increase in the production of reactive oxygen species (ROS) and thiobarbituric acid reactive substances (TBARS), as well as a reduction in the activity of superoxide dismutase (SOD) and catalase [73]. Additionally, AgNPs synthesized using *Endostemon viscosus* also showed potential use in natural pest control and as a source of new therapeutic agents. The biosynthesized AgNPs demonstrated potential larvicidal efficacy against *Ae*. *aegypti*, and the antibacterial activity also showed a maximum zone of inhibition for *Staphylococcus aureus* and *Escherichia coli*. The larvicidal effect was due to photocatalytic dye degradation activity, while the antibacterial activity was due to inhibition of the antioxidant effect [114].

Likewise, NPs derived from microorganisms also exhibit unique physicochemical properties that enhance their insecticidal efficacy. For instance, NPs synthesized from the endophytic fungus *Alternaria macrospora* demonstrated a strong ovicidal effect and the highest mortality rate in *Ae*. *aegypti* larvae, from the first- to fourth-instar stages, by inhibiting acetylcholinesterase activity [115]. Essential oil blends act as both repellents and toxic agents against disease-transmitting mosquitoes. For instance, Kamaraj et al. (2023) reported that essential oils were significantly more toxic to the larvae and pupae of *Cx*. *quinquefasciatus*, *An*. *stephensi*, and *Ae*. *aegypti* and also affected oviposition activity indices [116].

### 1.1. Aim of the Review

This systematic review aimed to consolidate existing knowledge on the use of MNPs in controlling mosquito vectors and vector-borne diseases. It assessed their efficacy, mechanisms of action, and potential integration into current vector control programs. By summarizing current research, the review offered a comprehensive understanding of MNPs' role in mosquito-borne disease control and highlighted areas for future research and application.

### 1.2. Objectives of the Review

  1.2.1. To assess the effectiveness of various MNPs in controlling mosquito populations, focusing on their impact on mortality, reproduction, and behavior.
  1.2.2. To explore the biological and chemical mechanisms through which MNPs affect mosquito vectors, emphasizing their insecticidal and repellent properties.
  1.2.3. To analyze how MNPs are integrated into existing mosquito control strategies, whether as standalone solutions or as supplements to conventional methods.
  1.2.4. To highlight the current research limitations and identify key areas where further studies are needed to optimize the use of MNPs in mosquito control.

### 1.3. Review Questions

  1.3.1. What were the different types of MNPs studied for mosquito vector control, and how effective were they in reducing mosquito populations?
  1.3.2. What were the primary mechanisms through which MNPs exerted their effects on mosquito vectors, and how did these mechanisms vary by nanoparticle type?
  1.3.3. How could MNPs be integrated into current mosquito control programs, and what challenges existed in their practical application?
  1.3.4. What were the potential risks and environmental impacts of using MNPs for mosquito control, and how could these be mitigated?
  1.3.5. What were the key gaps in the current research on MNPs in vector control, and what future directions should have been pursued to enhance their effectiveness and sustainability?

## 2. Methods

**Figure 1:**
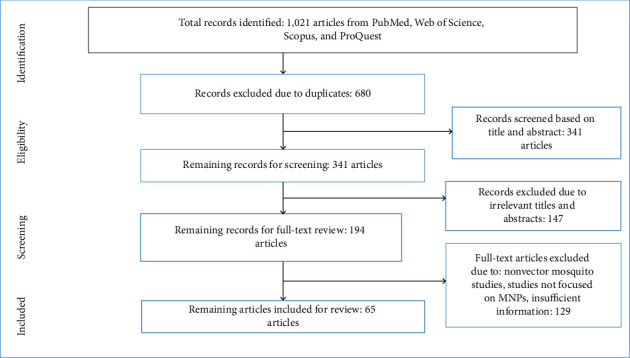
The flowchart of search and selection of article for review of mosquitocidal activity of metal NPs against mosquito: *Anopheles*, *Aedes,* and *Culex* mosquitoes.

This review examines the potential of MNPs to target different stages of mosquitoes from the genera *Anopheles*, *Culex*, and *Aedes*, following the Preferred Reporting Items for Systematic Reviews and Meta-Analysis (PRISMA) guidelines (Figure 1 and Supporting file 1: Table S1).

### 2.1. Search Strategy

A comprehensive search was conducted using global electronic databases (PubMed, Web of Science, Scopus, and ProQuest) between July 30 and August 15, 2022. The search terms included “Metal-based nanoparticles,” “Nanoparticles toxicity,” “Mosquito control,” “Larvicidal,” and “Nanomaterials in mosquito control” used in combination with Boolean operators like “OR” and “AND.”

### 2.2. Article Eligibility

The eligibility criteria for this systematic review were designed to include relevant studies addressing the impact of MNPs on mosquito vectors. Only peer-reviewed articles, along with randomized controlled trials, observational studies, and laboratory-based experimental studies, were considered. Studies have included mosquito species known as vectors of different diseases, such as *Aedes* species, *Anopheles* species, and *Culex* species. Eligible studies included reports on outcomes such as toxicity, mortality rates, behavioral changes, repellent effects, or breeding inhibition in mosquito vectors. Additionally, only studies published in English were included to ensure full analysis and the timeframe for inclusion was limited to studies published from the year 2011–2021 after the latest advancements in nanoparticle technology. In addition, the effect of biologically synthesized NPs against mosquitoes includes type and source of NPs, NP size (1–100 nm), mosquito larvae species tested exposure time, and LC50 and LC90 values, while reviews, meta-analyses, studies with insufficient data, unavailable full texts, gray literature, reviews, and congress abstracts were excluded.

### 2.3. Study Selections

The study selection process was carried out in two stages: The initial screening involved reviewing titles and abstracts of articles retrieved from the database searches. Studies were included if they met the eligibility criteria outlined above. Articles that were irrelevant (e.g., nonvector mosquito studies, studies not focused on MNPs, or those published in languages other than English) and studies with insufficient information were excluded at this stage. Whereas the studies passed the initial screening, the full texts were retrieved and examined in detail. The studies were assessed for methodological quality, relevance to the review's objective, and whether they provided sufficient data regarding the impact of MNPs on mosquito vectors. Studies that did not provide specific outcomes related to mosquito control or lacked data on MNP toxicity or efficacy were excluded. Data from studies on the mechanisms by which NPs affect mosquitoes were extracted.

### 2.4. Quality Assessment

Two authors assessed the quality of each original article using a standard quality assessment tool adapted from the JBI Critical Appraisal Checklist. Any disagreements were resolved through discussion or consultation with additional authors. Each study was assessed for potential biases, including methodological weaknesses and reporting inconsistencies. In cases where studies reported conflicting results, the review sought to explain these discrepancies through potential factors such as nanoparticle concentration, mosquito species, or experimental conditions.

### 2.5. Data Extraction and Presentation

The data were collected from the results of individual studies to determine general findings related to the potential of MNPs in mosquito control. Data on NP types, plant species, and mosquito larvae species tested exposure time, LC_50_, and LC_90_ values were extracted from the texts, tables, and figures of original studies and recorded in an Excel spreadsheet. Frequencies and percentages of data were calculated and presented.

## 3. Results

### 3.1. Characteristics of Original Articles

A total of 1021 articles were initially identified across multiple global databases (PubMed, Web of Science, Scopus, and ProQuest). After removing 680 duplicates, 341 articles remained for title and abstract screening. Of these, 147 were excluded due to irrelevant titles or abstracts, or because they were not published in English. Subsequently, 194 full-text articles were assessed for eligibility. From this set, 129 articles were excluded for reasons such as insufficient information, focusing on nonvector mosquito studies or lack of relevance to MNPs. The remaining 65 articles met the eligibility criteria and were included in the final review (Figure 1).

The highest number of publications (21.6%) occurred in 2015, followed by 10.8% each in 2014, 2017, and 2021. Most studies (85%) were conducted in India, with Egypt contributing 6%, and Iraq, China, and Latin America each contributing 3%.

The reviewed articles examined the mosquitocidal effects of NPs on different developmental stages of the genera *Anopheles*, *Culex*, and *Aedes*, which are common vectors of various diseases. The biosynthesis of NPs in these studies followed standard protocols involving plants and microorganisms [102, 117–119]. Larvicidal efficacy tests were conducted according to WHO guidelines for laboratory and field testing of mosquito larvicides [120].

**Figure 2:**
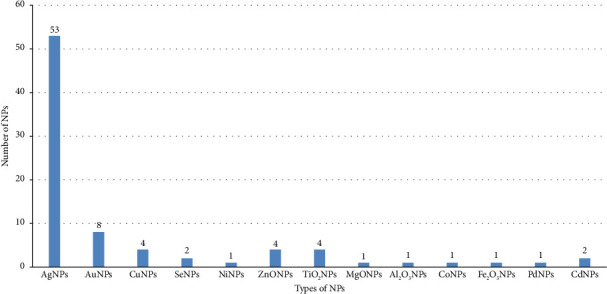
Types and quantity of NPs used for mosquitocidal activities tested in 2011–2022 years of interval.

Characterization of NPs in the studies was performed using UV spectroscopy, Fourier transform infrared spectroscopy (FTIR), scanning electron microscopy (SEM), X-ray diffraction (XRD), transmission electron microscopy, and other techniques. Even though each extraction method produces various shapes of NPs such as mono- or bimetallic, core/shell, or branched nanostructures, the reviewed articles reported that the majority (62%) of NPs have spherical shapes, while 32% are hexagonal, with average sizes of less than 100 nm. A total of 83 mosquitocidal efficacy tests were conducted. The majority (65.1%) used AgNPs, while gold nanoparticles (AuNPs) were the second most tested. These efficacy tests were performed on all stages of *Ae*. *aegypti*, *Ae*. *albopictus*, *An*. *stephensi*, *An*. *arabiensis*, *Cx*. *quinquefasciatus*, and *Cx*. *pipiens*. Notably, 24.1% (20/83) of the tests targeted all larval instars (I–IV) and pupae (Figure 2).

**Figure 3:**
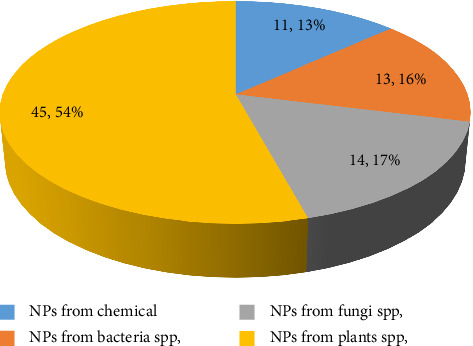
Proportion of biosynthesis of metal NPs from different parts of plants and different microorganisms for mosquitocidal activities against different stages of mosquito species.

The larvicidal activity of NPs was tested using extracts from various plant parts (leaves, seeds, flowers, and fruits) and different bacteria and fungi. AgNPs derived from *Azadirachta indica* bark and leaves were particularly lethal to *Cx*. *quinquefasciatus* first- and fourth-instar larvae, though less effective against pupae and adults. Similarly, fungal and bacterial-based NPs were effective and eco-friendly against *Ae*. *aegypti*, *Ae*. *albopictus*, *An*. *stephensi*, *An*. *arabiensis*, *Cx*. *quinquefasciatus*, and *Cx*. *pipiens* (Figure 3 and Supporting file 2: Table S2).

**Figure 4:**
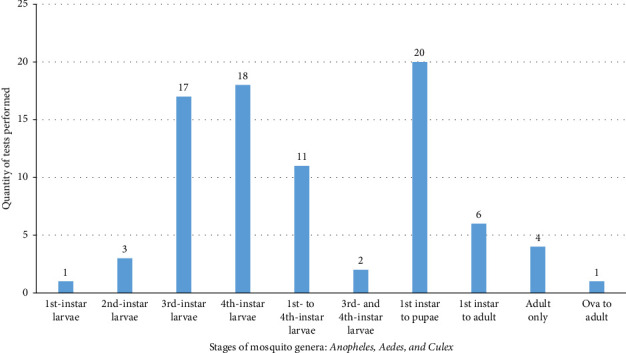
Proportion of different stages of *Anopheles*, *Culex*, and *Aedes* mosquitoes used to test the mosquitocidal activity of NPs.

This review also examined the mosquitocidal activities of different NPs across various mosquito developmental stages. The first-instar larvae to pupal stages were the most frequently tested, followed by the fourth- and third-instar larvae (Figure 4).

## 4. Discussion

### 4.1. Potential of Nanoparticle in the Fight Against Mosquito Vectors

Assessing the significance and efficacy of NPs in the control of mosquito vector populations, as well as their potential environmental implications, is of paramount importance. The application of NPs in vector control is an emerging field. Producing NPs is more cost-effective than traditional chemical methods due to its affordability, simplicity, environmental safety, and ease of scaling up. NP synthesis avoids the use of hazardous chemicals, high pressure, temperature, and energy consumption [121, 122]. Inorganic or metal-based NPs can be derived from various plant parts, microorganisms, and animal products, all of which exhibit insecticidal properties against different mosquito species and stages [104, 123]. However, other studies suggested that the implementation of nanotechnology in agriculture, particularly for pest control, needs to be carefully evaluated to ensure its benefits in the agricultural sectors in order to address public health concerns related to nanotoxicity [124, 125].

This review analyzed and evaluated 65 papers, revealing a total of 83 mosquitocidal efficacy tests conducted on MNPs. Among these, 54.2% were biosynthesized from plant products and 35.8% from microorganisms. Notably, the majority of plant-derived NPs were synthesized using *Azadirachta indica*. Similarly, of the microorganism-based NPs, 51.9% were from fungi and 48.1% from bacteria. Green synthesis of MNPs has gained attention due to its nontoxicity, accessibility, and consistent NP preparation methods, making plant-based NPs efficient for mosquito control [84, 126–132]. Plant-origin NPs are preferred for their minimal harm to nontarget organisms and biodegradability, positioning medicinal plants as alternative mosquito control agents [84, 127, 132].

Metal-based NPs derived from various plant parts and microbial species have shown effective larvicidal, pupicidal, and adulticidal properties [133–137]. The efficacy tests included 24.1% on all larval and pupal stages, 21.7% on fourth-instar larvae, 20.7% on third-instar larvae, and 15.9% on all instar larvae. Additionally, ovicidal, adulticidal, and oviposition deterrence effects were noted, likely due to NPs' ability to penetrate the exoskeleton and impact the oxidative processes of target mosquitoes [138].

Silver NPs (AgNPs) are the most studied for their broad-spectrum insecticidal activities. AgNPs from *A*. *indica* demonstrated 100% mortality of various mosquito species at low concentrations. In support of this finding, other studies showed AgNPs of *A*. *indica* have the potential to be used in the development of safe and eco-friendly larvicide for the control of mosquito larvae [139–141]. Similarly, AgNPs from *Mimusops elengi* effectively targeted *An*. *stephensi* and *Ae*. *albopictus*. Microbial AgNPs, like those from *Streptomyces anulatus* and *Listeria monocytogenes*, showed strong effects against all stages of *An*. *stephensi* [142, 143]. These NPs induce cytotoxicity and genotoxicity through oxidative stress [104, 144].

AgNPs derived from *A*. *indica* plant have been extensively researched and demonstrated effective mosquitocidal effects against various stages of *Cx*. *quinquefasciatus*, *Ae*. *aegypti*, and *An*. *stephensi*, resulting in 100% mortality within 15 min–24 h at concentrations ranging from 0.006 to 42 ppm. The application of *A*. *indica* plant in disease treatment and prevention is attributed to its high antioxidant properties, which modulate cell signaling pathways and exhibit anti-inflammatory effects through the regulation of proinflammatory enzyme activities [145]. Similarly, another study reported that *A*. *indica* is being researched in fields, such as dentistry, food safety, bacteriology, mycology, virology, and parasitology, and has been found safe for humans and environmentally friendly [146]. Conversely, another study suggested that certain compounds extracted from *A*. *indica* effective for pest control but might have detrimental effects on the liver and kidneys [147].

Similarly, multiple studies have reported the efficacy of AgNPs against *Ae*. *aegypti*, *Anopheles stephensi*, and *Cx*. *quinquefasciatus* larvae. For instance, AgNPs synthesized from *Laureliopsis philippiana* showed LC_50_ values of 29.596 and 23.946 μg/mL against *Ae*. *aegypti* and *An*. *stephensi*, respectively, after 12 h of exposure [148]. In addition, AgNPs from *Cassia roxburghii* demonstrated LC_50_ values of 26.35, 28.67, and 31.27 μg/mL against *An*. *stephensi*, *Ae*. *aegypti*, and *Cx*. *quinquefasciatus*, respectively [149].

Interestingly, some studies reported even lower LC_50_ values, indicating higher efficacy. AgNPs synthesized from *Chrysanthemum indicum* exhibited LC_50_ values ranging from 5.07 to 35.05 ppm against different larval instars of *An*. *stephensi* [150]. Moreover, AgNPs from *A*. *indica* exhibited remarkably low LC_50_ values of 0.006 and 0.047 mg/L against *Ae*. *aegypti* and *Cx*. *quinquefasciatus*, respectively [151].

On the other hand, AgNPs synthesized from bacteria such as *Streptomyces anulatus* and *Listeria monocytogenes* have shown mosquitocidal effects against *An*. *stephensi*, with a minimum LC_50_ and LC_90_ values indicating potent effects. These findings were further supported by other study findings [142, 152]. Researches on nanobiotechnology and microbial synthesis of NPs have emphasized their strong mosquitocidal activities, attributed to the shape, charge, and size of AgNPs. These properties contributed to cytotoxicity and genotoxicity by inducing oxidative stress in insect tissues [106, 138, 153].

Similarly, AgNPs derived from various fungi and bacteria, including *Beauveria bassiana*, *Chrysosporium keratinophilum*, *Lecanicillium lecanii*, *S*. *anulatus*, *Bacillus subtilis*, *L*. *monocytogenes*, and *Bacillus amyloliquefaciens*, demonstrated a strong larvicidal activity against *An*. *stephensi*, *Ae*. *aegypti*, and *Cx*. *quinquefasciatus*. These findings highlighted the potential of these AgNPs for diverse applications, including the control of pathogenic fungi and bacteria, the treatment of cancer cells and viruses, and their roles in providing larvicidal and insecticidal activities [154, 155].

The mechanism underlying the larvicidal effect of AgNPs involves significant degradation to the larvae's hindgut, epithelial cells, midgut, and cortical areas. This effect was achieved through the inhibition of the acetylcholine esterase, α- and β-carboxylesterases, and phosphatase activities in the 4^th^-stage larvae [111–113]. This induced a redox imbalance in the larvae, marked by an increase in the production of ROS and TBARS, as well as a reduction in the activity of SOD and catalase [73, 156]. This damage disrupts key physiological functions, enhancing the NPs' effectiveness in larval control. These eco-friendly NPs present a promising alternative to traditional synthetic insecticides for mosquito control, potentially minimizing environmental impact and the risk of developing insecticide resistance [157].

Likewise, AuNPs emerge as the second most researched and promising option for vector control. AuNPs derived from various fungi species, including *C*. *keratinophilum*, *Entomophthora culicis*, *A*. *niger*, and *V*. *lecanii*, demonstrated potent larvicidal activity against different larval stages of *An*. *stephensi*, *Cx*. *quinquefasciatus*, and *Ae*. *aegypti* [158–161]. Similarly, tests conducted at varying concentrations and exposure times highlighted their strong insecticidal efficacy. In addition, historical medicinal uses of novel metals like Ag and Au underscore their significance in traditional and contemporary medical practices, enhancing the appeal of AuNPs synthesized from fungi as a swift, eco-friendly approach to mosquito control [106, 160–163].

On the other hand, AuNPs synthesized from plant sources such as *Citrus limon*, *Artemisia vulgaris*, and *Anthocephalus cadamba* displayed strong larvicidal effects against various mosquito species. Interestingly, other plant sources have also shown promise in synthesizing NPs with mosquitocidal properties. For instance, AuNPs and AgNPs biosynthesized from the bark extracts of *Cinnamomum zeylanicum* demonstrated potent larvicidal activity against *Cx*. *quinquefasciatus* and *An*. *Stephensi*, with *An*. *stephensi* being more susceptible [164]. The study using *Citrus limon* leaf extract to synthesize Au-Pd bimetallic nanoparticles (BNPs) showed effective larvicidal activity against *An*. *stephensi* and *Ae*. *aegypti* mosquito larvae [165]. The LC_50_ values obtained indicated toxicity to mosquito larvae while having no lethal effects on nontarget aquatic predators.

Similarly, another study reported that various MNPs caused significant pharmacological effects through the induction of oxidative stress [166]. For example, AuNPs synthesized using *Artemisia vulgaris* L. leaf extract showed significant larvicidal effects against *Ae*. *aegypti* larvae, with LC_50_ values of 62.47 ppm and 43.01 ppm for 3rd- and 4th-instar larvae, respectively, after 24 h of exposure [167]. Likewise, *zein biopolymer*–synthesized AuNPs (Ze-AuNPs) demonstrated potent larvicidal activity against *Ae*. *aegypti*, with an LC_50_ of 6.81 mg/L [168]. Additionally, essential oils from plants like *Zanthoxylum limonella* and *Curcuma zedoaria* showed significant larvicidal effects against *An*. *dirus* and *Ae*. *Aegypti* [169].

Interestingly, some studies have found that plant-synthesized AuNPs can be more effective than their corresponding plant extracts. For example, marigold flower petal-mediated CdNPs demonstrated 100% mortality against mosquito larvae after 72 h of incubation at a concentration of 10 ppm [170]. This suggests that NP formulations may enhance the larvicidal efficacy of plant-derived compounds. In conclusion, plant-synthesized NPs, particularly those derived from *Citrus limon* and other sources, exhibit strong larvicidal activity against various mosquito species. This eco-friendly approach offers a promising avenue for mosquito control, potentially reducing the reliance on synthetic pesticides.

Copper nanoparticles (CuNPs), derived from *Metarhizium robertsii* and *Tridax procumbens*, demonstrated significant larvicidal efficacy against different mosquito species. CuNPs synthesized using the whole-cell biomass of *Fusarium proliferatum* (YNS2) exhibited significant larvicidal activity against *An*. *stephensi*, *Ae*. *aegypti*, and *Cx*. *quinquefasciatus*. The LC_50_ values were 81.34 μg/mL, 39.25 μg/mL, and 21.84 μg/mL for *An*. *stephensi*, *Ae*. *aegypti*, and *Cx*. *quinquefasciatus*, respectively [171]. This indicates that CuNPs were most effective against *Cx*. *quinquefasciatus* larvae. As for *Tridax procumbens*, the context mentions the synthesis of copper oxide nanoparticles (CuONPs) using its leaf extract. These CuONPs were tested for larvicidal activity against *Ae*. *aegypti*, but specific efficacy data are not provided [172].

Similarly, another study reported that CuNPs synthesized from *Astragalus sinicus* exhibited strong acaricidal, larvicidal, and repellent activities against both adults and larvae of *Hyalomma anatolicum* [173]. *H*. *anatolicum* is the most prevalent ticks infesting cattle in Saudi Arabia. Likewise, *M*. *robertsii* has been reported as the most effective entomopathogenic fungus [174]. The mode of action primarily involves rapid disruption of membrane integrity, leading to oxidative damage and inhibiting juvenile mosquito stages [175–177]. Additionally, CuNPs synthesized using *Wrightia tinctoria* extract exhibited larvicidal activity against *Ae*. *aegypti* [178].

Zinc oxide nanoparticles (ZnONPs) synthesized from seaweed sources and plant extracts have demonstrated significant larvicidal effects against medically important vectors. Multiple studies have reported the efficacy of these green-synthesized ZnONPs against mosquito larvae, particularly *Ae*. *aegypti* and *Cx*. *quinquefasciatus*. Seaweed-derived ZnONPs showed potent larvicidal activity against *Ae*. *aegypti*, with 100% mortality of fourth-instar larvae observed at 50 μg/mL concentration within 24 h [179]. ZnONPs prepared from *Brassica oleracea* var. botrytis leaf extract demonstrated larvicidal activity against *Cx*. *quinquefasciatus* fourth-instar larvae, with LC_50_ and LC_90_ values of 76.03 and 190.03 ppm, respectively [180]. Furthermore, *Murraya koenigii* berry extract–based ZnONPs showed high larvicidal activity against *Cx*. *quinquefasciatus* larvae, with LC_50_ and LC_90_ values of 2.1 and 12.1 μg/mL, respectively [181]. Additionally, ZnONPs synthesized using *Momordica charantia* leaf extract exhibited potent larvicidal effects against *An*. *stephensi* and *Cx quinquefasciatus* larvae, with LC_50_ values of 5.42 and 4.87 mg/L, respectively [83].

Studies also highlighted the damaging impact of ZnONPs on mosquito midgut cells, emphasizing their potential in controlling vector-borne diseases [182]. Similarly, another study reported that combining ZnO and titanium oxide nanoparticles (TiO_2_NPs) has a synergistic insecticidal effect against *Bactericera cockerelli* Sulc. (Hemiptera: Triozidae) when tested on tomato (*Solanum lycopersicum*) [183].

TiO_2_NPs from various plant sources demonstrated strong larvicidal effects, with their synergistic activity in combination with ZnONPs enhancing efficacy. Likewise, another study offers groundbreaking evidence on the potential use of *Desmostachya bipinnata*–mediated TiO_2_NPs for controlling mosquito vectors (*Ae*. *aegypti*) and managing agricultural pests (*Spodoptera litura*) and it also caused acute toxicity in nontarget organisms [184]. The presence of ROS played a pivotal role in disrupting larvae cells, leading to mortality [183]. Several studies have demonstrated the efficacy of various NPs against ticks and other parasites. For instance, TiO_2_NPs synthesized using *Mangifera indica* leaf extract showed acaricidal activity against *Hyalomma anatolicum anatolicum* larvae with an LC_50_ value of 33.17 mg/L [185]. Similarly, TiO_2_NPs synthesized from *Solanum trilobatum* leaf extract exhibited acaricidal activity against *H*. *a*. *anatolicum* with an LC_50_ value of 4.11 mg/L [186].

Selenium nanoparticles (SeNPs) synthesized from different sources exhibited varying levels of larvicidal potency, with factors like stability, size, and shape influencing their toxicity [187–189]. The biofabricated SeNPs using cell-free extract of *Xenorhabdus cabanillasii* (XC-SeNPs) showed dose-dependent larvicidal effects against *Ae*. *aegypti* larvae, with LC_50_ and LC_90_ values of 79.4 and 722.4 ppm, respectively [190]. Similarly, green-synthesized AgNPs from plant extracts exhibited effective larvicidal activity against *An*. *stephensi*, even at low concentration [191].

Similarly, another study demonstrated that pyrazolopyrimidine SeNP derivatives exhibit significant larvicidal bioefficacy against *Cx*. *pipiens* [192]. Selenium ion binding affinity to the 40S ribosomal protein S7 was revealed by a molecular docking analysis of mosquito ribosomal proteins. Additionally, SeNPs have demonstrated potent antibacterial, anti-biofilm, antioxidant, and insecticidal properties [193]. Interestingly, the shape and size of nanoparticles can significantly influence their biological activity. For instance, cubic-like SeNPs synthesized using folic acid–gallic acid–N,N,N-trimethyl chitosan (FA–GA–TMC) as a stabilizer showed good anticancer efficacy and cellular uptake against breast cancer cells while exhibiting low toxicity to normal cells [194]. This suggests that the shape and size of nanoparticles can be controlled to enhance their biological effects. Magnesium oxide nanoparticles (MgO-NPs) showed promise in controlling *An*. *stephensi*, with their repellent efficacy providing protection against adult mosquitoes [195]. The biogenic MgO-NPs fabricated using metabolites from *Penicillium chrysogenum* exhibited high efficacy against different larval instars and pupae of *An*. *stephensi*, with LC_50_ values ranging from 12.5 to 15.5 ppm for larvae and 16.5 ppm for pupae [196]. More importantly, when applied at a concentration of 5 mg/cm2, MgO-NPs showed remarkable repellent activity against adult *An*. *stephensi* mosquitoes, providing 100% protection for 150 min and 67.6% ± 1.4% protection for 210 min [196].

Interestingly, while MgO-NPs show promise, other natural compounds have also demonstrated repellent properties against *An*. *stephensi*. For instance, essential oils from plants like clove (*Syzygium aromaticum*) provided protection for up to 63 min at a concentration of 0.5% (v/v) [197]. Additionally, leaf extracts from plants such as *Solanum trilobatum* and *Ervatamia coronaria* have shown significant repellent activities against *An*. *stephensi* [198, 199]. Similarly, another study reported that MgO-NPs have an effective insecticidal effect on the larvae of *An*. *stephensi* [200].

Iron and iron oxide nanoparticles (Fe and Fe_2_O_3_NPs) displayed larvicidal and pupicidal effects against *Cx*. *quinquefasciatus*, with chemically synthesized Fe_2_O_3_NPs showing enhanced efficacy. Similarly, Fe and Fe_2_O_3_NPs derived from *Cassia auriculata* flower showed larvicidal and pupicidal activity against *Cx*. *quinquefasciatus* [201]. Interestingly, Fe_2_O_3_NPs synthesized using plant extracts also exhibited potent larvicidal activity against *Cx*. *quinquefasciatus*. For instance, Fe_2_O_3_NPs produced using *Pterolobium hexapetalum* leaf extract showed LC_50_ and LC_90_ values of 2.437 and 10.639 mg/mL, respectively [202]. This suggests that green synthesis methods can potentially enhance the larvicidal efficacy of Fe_2_O_3_NPs. Additionally, chemically synthesized Fe_2_O_3_NPs showed higher toxicity, with LC_50_ values ranging from 4.5 ppm for first-instar larvae to 22.1 ppm for pupae. In comparison, green-synthesized FeONPs had LC_50_ values between 20.9 ppm for first-instar larvae and 43.7 ppm for pupae of *Cx. quinquefasciatus* [203].

Nickel nanoparticles (NiNPs) demonstrated excellent larvicidal activity against *An*. *subpictus* and *Cx*. *quinquefasciatus*, underscoring their potential in vector control [204, 205]. Another study demonstrated that NiNPs synthesized using extracellular metabolites of *B*. *sphaericus* have been effective in controlling vector-borne diseases [206, 207]. The study found that the bacterial metabolites acted as reducing and capping agents in the synthesis process, resulting in spherical to oval-shaped NiNPs with an average diameter of 23 ± 2.00 nm [206]. The NiNPs showed high larvicidal activity against fourth-instar larvae of *An*. *subpictus and Cx*. *quinquefasciatus* at LC_50_ values of 6 ppm which resulted in 100% mortality of larvae (IV) stage of *An*. *subpictus* and *Cx*. *quinquefasciatus* after exposure for 24 h at 10 ppm. The findings revealed that synthesized NiNPs had excellent larvicidal activities. A review regarding the biogenesis and application of NiNPs reported that green-synthesized nickel can be used for the application of catalytic, antimicrobial, cytotoxicity, and antioxidant activity [208].

Interestingly, integration of AgNPs and TiO_2_NPs has also shown excellent larvicidal activity against these vector species. For instance, AgNPs synthesized using *Eclipta prostrata* extract demonstrated high efficacy against *Cx*. *quinquefasciatus* and *An*. *subpictus* [209], while TiO_2_NPs synthesized with *Vitex negundo* leaf extract exhibited strong larvicidal activity against the same species [210].

Palladium NPs (PdNPs) and cadmium NPs (CdNPs) exhibited significant larvicidal effects against various mosquito species. PdNPs synthesized using *Melia azedarach* leaf extract showed significant larvicidal activity against *Ae*. *aegypti*, with LC_50_ and LC_90_ values of 27.36% and 52.50%, respectively [211]. Similarly, PdNPs synthesized from *Cymodocea serrulata* showed larvicidal effects against *An*. *stephensi*, with LC_50_ and LC_90_ values of 4.010 and 80.827 μg/mL for 1st-instar larvae [212]. Interestingly, CdNPs synthesized using marigold flower petal extract achieved 100% mortality against mosquito larvae after 72 h of incubation at a concentration of 10 ppm [170]. Additionally, CdNPs synthesized from plant extracts exhibited potent larvicidal effects against *An*. *albopictus*, underscoring their potential as biological control agents [213]. Despite the promising results, it is crucial to study the nontarget effects of NPs before their widespread application [214].

Cobalt nanoparticles (CoNPs) and PdNPs synthesized from various sources exhibited promising larvicidal effects against mosquito larvae, suggesting their utility in vector management [215]. In support of this, PdNPs from *Melia azedarach* leaf extract exhibited an LC_50_ of 27.36% against *Ae*. *aegypti* larvae. Meanwhile, some of the papers discuss the antimicrobial properties of CoNPs, which could potentially be relevant to mosquito-borne disease research [216]. For example, one study reported on the antibacterial and antifungal activities of green-synthesized CoNPs, noting that CoNPs showed good antimicrobial activity against tested microbes [217]. Similarly, another study mentioned that CoNPs demonstrated remarkable antimicrobial activity against common bacterial and fungal pathogens [216]. The antimicrobial properties of CoNPs could be explored in future research related to vector control or pathogen inhibition in the context of mosquito-borne diseases.

### 4.2. Environmental Impact and the Possible Consequences of Nanoparticles

Several MNPs, including Ag, Au, ZnO, TiO_2_, CuO, and others, have demonstrated significant potential in managing mosquito populations. Nonetheless, a thorough assessment of the environmental ramifications associated with these NPs is necessary to promote their sustainable application.

Although AgNPs exhibit remarkable efficacy in controlling mosquito larvae, they simultaneously present considerable threats to aquatic ecosystems. Research has indicated that AgNPs can be harmful to a range of aquatic life forms, such as fish, invertebrates, and algae [218, 219]. The toxic effects are primarily due to the release of silver ions (Ag^+^), which can induce oxidative stress and cause cellular injuries, ultimately resulting in the mortality of these organisms [220, 221]. Additionally, another investigation has highlighted the tendency of AgNPs to accumulate in soil environments, which could potentially disrupt microbial populations crucial for processes like nutrient cycling and the decomposition of organic matter. Such disruptions may lead to declines in soil fertility and shifts in ecosystem dynamics [222].

The environmental impact of AuNPs is a multifaceted issue that has not been extensively covered. However, some inferences can be drawn from the studies' focus on the synthesis, properties, and applications of AuNPs. For instance, the use of *Pseudomonas aeruginosa* for the biosynthesis of AuNPs suggests a biological route that may offer an environmentally benign alternative to chemical synthesis methods [223]. Conversely, the study of AuNPs' influence on somatic embryogenesis in *Arabidopsis thaliana* indicates that NPs can interfere with biological processes, which could have ecological implications if such interactions occur in the environment [224]. Other study also has indicated that AuNPs are capable of triggering oxidative stress, inflammation, and genotoxic consequences in various aquatic species, including fish, algae, and invertebrates [218]. AuNPs have been demonstrated to influence plant growth and metabolic processes, which may result in diminished agricultural output and changes to plant physiological responses [225]. There is also a concern about the potential transfer of AuNPs into the food chain, which could have unknown health effects on higher trophic levels, including humans [224, 226]. The unintended discharge of AuNPs into the environment has the potential to disturb soil microbial ecosystems, which play a crucial role in the cycling of nutrients and the overall health of soil. Such releases could lead to extensive pollution of aquatic systems, terrestrial soils, and possibly the atmosphere, with unpredictable effects on global ecological systems [227].

The environmental impact of ZnONPs is a growing concern due to their widespread use and potential ecotoxicological effects. Studies have shown that ZnONPs can enter aquatic ecosystems, leading to adverse effects on aquatic organisms such as common carp, with implications for ecosystem health and human safety [228, 229]. Other studies also have demonstrated harmful effects on certain species, such as inhibiting seedling radicle growth in *Capsicum annuum* L. [230]. The potential consequences of prolonged exposure to ZnONPs at varied concentrations remain under-investigated, with studies indicating that ZnONPs can cause histopathological changes in fish and affect the biology of model organisms like *Galleria mellonella* [228, 231]. Furthermore, ZnONPs may bioaccumulate in various aquatic species, which could result in biomagnification throughout the food web. Such processes pose significant risks to the health of ecosystems and the diversity of species within them [229, 232].

TiO_2_NPs have been identified as having potential environmental risks due to their widespread use in various industrial and commercial products [233]. The environmental impact of TiO_2_NPs includes their persistence in the environment, as they exhibit very low dissolution rates in both biological and environmental media, suggesting that they will remain largely unchanged over long periods [234, 235]. This persistence raises concerns about their long-term health effects and environmental impact. Additionally, TiO_2_NPs have been shown to induce oxidative stress, leading to cell damage, inflammation, genotoxicity, and adverse immune responses in various organisms [236–238]. These effects are influenced by the physical and chemical properties of the nanoparticles, such as size and surface reactivity, and are exacerbated by long-term exposure [238, 239].

CuNPs pose a risk to aquatic life forms such as fish, invertebrates, and algae. The hazardous natures of these NPs are primarily due to their tendency to dissolve into copper ions (Cu^2+^), which can disrupt crucial biological mechanisms. For example, Cu^2+^ ions can interfere with the respiratory functions of fish by binding to gill proteins, which may induce hypoxic conditions and, in extreme instances, lead to death [240]. Prolonged exposure to high concentrations of CuNPs has been found to inflict damage upon the liver and kidneys, in addition to producing neurological impairments in both wildlife and human populations [241]. Similarly, CuONPs can induce oxidative stress, hinder enzymatic activity, and contribute to mortality among fish, invertebrates, and soil-dwelling organisms [221]. Like other MNPs, CuONPs are prone to accumulating in both soil and aquatic environments, which can lead to persistent contamination issues. Such accumulation may compromise soil fertility and disrupt the functionalities of aquatic ecosystems over the long term [241, 242].

Selenium plays a crucial role as a micronutrient for a wide range of organisms; however, SeNPs can pose toxicity risks when present in elevated concentrations. Aquatic life forms, such as fish, algae, and invertebrates, are particularly sensitive to the toxic effects of selenium. Increased concentrations of SeNPs in aquatic ecosystems can result in their accumulation within these organisms, which may in turn lead to reproductive challenges, physical deformities, and increased mortality rates, particularly affecting fish and amphibians [243]. Furthermore, prolonged exposure to SeNPs may induce oxidative stress and cause cellular harm within aquatic species [244, 245]. Additional research indicated that long-term selenium exposure in humans can also result in toxicity, referred to as selenosis, which manifests through various symptoms including hair and nail loss, neurological impairment, and gastrointestinal issues [246].

MgO-NPs possess the potential to harm aquatic life, impacting a range of organisms from algae to fish and invertebrates. The chief cause of this toxicity arises from the liberation of magnesium ions (Mg^2+^) from the NPs, which can disrupt key biological functions. Increased concentrations of Mg^2+^ may induce oxidative stress, cause ionic imbalances, and modify metabolic activities in aquatic species, while also potentially contributing to human health concerns, such as gastrointestinal issues and disruptions in electrolyte balance [247]. Research has indicated that exposure to MgO-NPs can hinder growth and lead to developmental defects in fish embryos [248]. The long-term implications of bioaccumulation within aquatic environments remain inadequately understood; however, they possess the potential to create ripple effects that impact ecosystem health [249]. Moreover, heightened levels of Mg^2+^ can significantly modify the chemistry of soil and water, potentially influencing the availability of other vital nutrients and the well-being of various organisms [250].

NiNPs pose a risk to various aquatic life forms, including fish, algae, and invertebrates. The primary cause of their toxicity lies in the release of nickel ions (Ni^2+^), which disrupt critical biological functions. Increased concentrations of Ni^2+^ can disturb ionic equilibrium, hinder enzymatic functions, and induce oxidative stress in both aquatic organisms and terrestrial animals [251]. Research indicates that exposure to NiNPs can result in stunted growth and oxidative harm in fish and algae [252, 253]. Furthermore, the bioaccumulation of nickel can generate toxic effects in higher level consumers, such as predatory birds and mammals that feed on fish, potentially resulting in reproductive and developmental complications [254]. Moreover, prolonged exposure to nickel can lead to various health concerns, including respiratory illnesses, skin disorders, and damage to the kidneys [255].

Similarly, studies suggested that PdNPs also can pose a risk to various aquatic species, including fish, algae, and invertebrates. This toxicity is largely due to the liberation of palladium ions (Pd^2+^) from the nanoparticles, which exhibit greater solubility and bioavailability than their particulate counterparts, thereby amplifying their effects on both aquatic and terrestrial life [256, 257]. Research has indicated that PdNPs can adversely affect the growth and reproductive processes of algae, while also leading to developmental defects in fish embryos [256, 258, 259]. The prolonged effects of palladium accumulation within aquatic environments are under ongoing investigation, yet they may bring about considerable consequences for ecosystem integrity and biodiversity [260]. Furthermore, finding has highlighted concerns regarding the accumulation of palladium in the tissues of edible plants, which raises issues related to food safety and potential risks to human health [261].

Biosynthesized NPs, especially Ag and AuNPs, exhibit significant promise for use in mosquito control due to their stability and consistent morphology. To substantiate these initial findings, additional field studies are essential, accompanied by the formulation of best practice guidelines for the application of NPs in mosquito management. While MNPs have proven effective against numerous mosquito species and life stages, comprehensive investigations into their effects on nontarget organisms are imperative prior to their widespread deployment in vector management strategies. Understanding these effects is vital for responsible management and to mitigate potential environmental impacts.

### 4.3. Future Recommendations for Nanoparticle Application

Some NPs triggered contrary effects on nontarget organisms like *Anisops bouvieri*, *Diplonychus indicus*, *Poecilia reticulata*, and *Gambusia affinis* which are natural enemies predating mosquito larvae and pupae [165, 213, 262, 263]. Mosquitocidal NP formulations like nanoemulsions and nanodispersions have a bright future and potential for developing safer and more effective mosquitocidal preparations for vector control, which potentially could result in revolutionary changes in this field.

Similarly, a few studies have reported that MNPs can have ecological impacts due to their persistence in the environment and effects on aquatic and terrestrial ecosystems. Additionally, researchers have highlighted potential health risks to humans and other nontarget organisms [264–267] and further assessment of the side effects of MNPs is recommended, particularly for those involved in their application and for communities living in treated areas. Promoting eco-friendly synthesis techniques using plant extracts, microorganisms, and other natural sources is crucial to refining synthesis methods for enhancing the efficacy and sustainability of MNPs [88, 268]. This approach helps to reduce the environmental footprint of MNP production. Additionally, developing scalable and cost-effective production methods is essential to facilitating the widespread use of MNPs in vector control programs.

Understanding the precise mechanisms by which MNPs exert their insecticidal effects on mosquito vectors is crucial. Several studies have demonstrated the interaction of MNPs at the cellular and molecular levels in mosquito larvae and adults [83, 108, 269–272]. This involves studying their effects on enzyme activity, cell membrane integrity, and oxidative stress. However, to mitigate the risk of mosquito resistance and nontarget toxicity, it is essential to rotate different types of MNPs, combine MNPs with other control methods, and conduct ongoing resistance monitoring [273–277].

We evaluate MNPs in diverse geographical regions with varying mosquito species and environmental conditions to gauge their broad-spectrum effectiveness. Expand field trials to confirm MNPs' efficacy under different environmental scenarios. Integrate MNPs with established vector control strategies like ITNs, larvicides, and biological controls to enhance overall effectiveness. To enhance the evaluation and regulatory approval of MNPs, it is crucial to establish standardized testing protocols that assess their efficacy and safety. Collaborating with regulatory agencies can streamline the approval process for new MNP-based products, ensuring their timely and safe integration into various applications.

Finally, conducting education campaigns to inform the public about the benefits and safety of using MNPs for mosquito control and planning and executing vector control measures to ensure acceptance and cooperation are important. This will increase public awareness and involve communities in the implementation of MNP-based vector control programs. These recommendations aim to guide future research and development efforts, ensuring that MNPs can be effectively and safely integrated into mosquito vector control strategies.

## 5. Limitation of the Study

The strength of the evidence is backed by a wide range of laboratory-based studies. However, there are limitations, including potential publication bias, since only studies published in English were included, possibly excluding relevant research in other languages. Furthermore, the variability in NP synthesis methods, dosages, and testing conditions across studies made direct comparisons challenging. The review also lacked long-term field data on the environmental impact and efficacy of MNPs in real-world applications, which may limit the generalizability of the findings.

## 6. Conclusions

This systematic review consolidates the potential of MNPs in mosquito vector control, emphasizing their effectiveness across various mosquito species and life stages. Key findings indicate that MNPs, particularly silver, gold, copper, and zinc oxide nanoparticles, show strong insecticidal effects, including larvicidal, pupicidal, and adulticidal properties. AgNPs are the most studied due to their broad-spectrum insecticidal activities. *A*. *indica*-derived AgNPs demonstrated 100% mortality of *Cx*. *quinquefasciatus*, *Ae*. *aegypti*, and *An*. *Stephensi* larvae. AgNPs from *M*. *elengi*, *L*. *philippiana*, and *C*. *roxburghii* further confirmed strong larvicidal activity. Microbial AgNPs from *S*. *anulatus* and *L*. *monocytogenes* also exhibited strong efficacy against *An*. *stephensi*. Similarly, AuNPs have also demonstrated strong insecticidal properties, particularly when synthesized from fungi (*C*. *keratinophilum*, *E*. *culicis*, *A*. *niger*, and *V*. *lecanii*). Studies confirmed potent larvicidal activity against *An*. *stephensi*, *Cx*. *quinquefasciatus*, and *Ae*. *aegypti*. Additionally, plant-based AuNPs from Citrus limon, *A*. *vulgaris*, and *A*. *cadamba* showed promising results. Notably, Au-Pd BNPs synthesized from Citrus limon leaf extract effectively targeted *An*. *stephensi* and *Ae*. *aegypti* larvae without harming nontarget aquatic species. CuNPs derived from *M*. *robertsii*, *T*. *procumbens*, and *F*. *proliferatum* exhibited strong larvicidal activity against *An*. *stephensi*, *Ae*. *aegypti*, and *Cx*. *quinquefasciatus*, with the latter being the most susceptible. Furthermore, ZnONPs synthesized from seaweed sources and plant extracts, such as *B*. *oleracea*, *M*. *koenigii*, and *M*. *charantia*, demonstrated high larvicidal efficacy. Similarly, TiO_2_NPs, when combined with ZnONPs, exhibit a synergistic larvicidal effect by inducing oxidative stress in mosquito larvae and disrupt the larval physiology. This leads to cellular damage and increased larval mortality. MgO-NPs have demonstrated potent repellent activity against An. stephensi, providing complete (100%) protection for up to 150 min. Ni, Pd, CdNPs: Nickel NPs (LC50 6 ppm) and PdNPs (LC50 4.01 μg/mL) showed high larvicidal activity. The mechanism of action involves oxidative stress, cytotoxicity, and genotoxicity, leading to the disruption of mosquito larvae physiological processes.

The synthesis of these nanoparticles, often derived from plant and microbial sources, has gained attention for its cost-effectiveness, environmental safety, and minimal toxicity to nontarget organisms. However, the environmental impact of MNPs remains a significant concern, with studies indicating potential toxicity to aquatic ecosystems, soil, and other nontarget organisms. These concerns underscore the need for further research into the long-term ecological consequences of MNP use, especially in real-world scenarios.

Finally, MNPs have emerged as a promising alternative to conventional chemical insecticides for mosquito control, offering significant benefits in terms of effectiveness and environmental safety. However, further studies are necessary to address gaps in understanding, particularly concerning their long-term environmental impact, toxicity to nontarget organisms, and field-level efficacy. Future research should concentrate on developing eco-friendly formulations and establishing regulatory frameworks to ensure the safe and sustainable use of MNPs in managing vector-borne diseases.

## Data Availability

The data that supports the findings of this study are available in the supporting information of this article.

## Nomenclature

IRS: Indoor residual spraying
ITNs: Insecticide-treated nets
LC: Lethal concentration
LF: Lymphatic filariasis
MNPs: Metal nanoparticles
ROS: Reactive oxygen species
WHO: World Health Organization

## Supporting Information

Supporting Information 1 Table S1: Preferred Reporting Items for Systemic reviews and Meta-Analysis (PRISMA) for Potential of Metal-Based Nanoparticles in the Fight Against Mosquito Vectors.

Supporting Information 2 Table S2: Summary of the efficacy of NPs synthesized from various microorganisms and plant species against different stages of mosquito genera (*Anophele*s, *Culex*, and *Aedes*).
