# Supplementary material for: A Global Systematic Review on the Potential of Metal-Based Nanoparticles in the Fight Against Mosquito Vectors
Source: J Trop Med. 2025 Jun 9;2025:2420073. doi: 10.1155/jotm/2420073 (PMC12170094; doi:10.1155/jotm/2420073)
Supplement: Supporting Information 2 — Table S2: Summary of the efficacy of NPs synthesized from various microorganisms and plant species against different stages of mosquito genera (Anopheles, Culex, and Aedes). [file 2420073.f2.doc]

*Supplementary file 2: Table S2: Summary of the efficacy of NPs synthesized from various microorganisms and plant species against different stages of mosquito genera (Anopheles, Culex, and Aedes.*

| NPs | NPs Size (nm) | Source of NPs | Mosquito species tested | Larva instar | Exposure time in hrs. | LC_50_ (ppm) | LC_90_ (ppm) | Reference (s) |
| --- | --- | --- | --- | --- | --- | --- | --- | --- |
| AgNP | 100 | *Moringa oleifera* (Plant seed *)* | *Ae. aegypti* | 1^st^-4^th^ /pupa | 48 | 10.2-16.7/21.2 | 23.1-35.7/42.6 | (1) |
| AgNP | 15-21 | *Lysinibacillus sphaericus* ( Bacteria *)* | *Cx. Pipiens* | 3^rd^ | 48 | 136-178 | 240-299 | (2) |
| AgNP | 15-20 | *Bacillus thuringiensis* ( Bacteria) | *Cx. Pipiens* | 3^rd^ | 48 | 55 | 184 |
| AgNP | 5.5-33 | *Ambrosia arborescens* (Plant leaf) | *Ae. aegypti* | 3^rd^ | 24 | 0.28 | 0.43 | (3) |
| AgNP | 14.01–21.02 | *Trichoderma atroviride* ( Fungi) | *Ae. aegypti* | 1^st^ | 7 | 0.9-4.8 | 1-6.3 | (4) |
| AgNP | 52 | *Pseudomonas* sp ( Bacteria) | *Ae. aegypti* | 3^rd^ | 24 | 0.5 | 1.3 | (5) |
| AgNP | 32 | *Cassia roxburghii* (Plant leaf ) | *An. stephensi,*  *Ae. aegypti,*  *Cx.quinquefasciatus* | 3^rd^  3^rd^  3^rd^ | 24  24  24 | 26.35  28.67  31.27 | 48.81  53.24  58.11 | (6) |
| AgNP | 13–34 | *Mukia maderaspatana (*Plant leaf) | *Ae.aegypti*  *Cx.quinquefasciatus.* | 4^th^  4^th^ | 24  24 | 0.2  0.09 | 0.7  0.5 | (7) |
| AgNP | 38 | *Chomelia asiatica* ( Plant leaf) | *An. stephensi,*  *Ae. aegypti,*  *Cx quinquefasciatus* | 3^rd^  3^rd^  3^rd^ | 24  24  24 | 17.95  19.32  20.92 | 33.03  34.87  37.41 | (8) |
| AgNP | 10- 14 | *Solanum mammosum* ( Plant fruits) | *Ae. aegypti* | 3^rd^ | 24 | 0.06 | 0.08 | (9) |
| AgNP | 37–61 | *Beauveria bassiana (* fungi) | *Ae. aegypti* | 1^st^/2^nd^ /3^rd^ /4^th^ | 24 | */*/0.06/0.06 | */*/1/1.09 | (10) |
| AgNP | 34 | *Dicranopteris linearis* ( Plant leaf) | *Ae. aegypti.* | egg/1^st^-4^th^/Pupa | 72 | NA/18.9-26.3/29.3 | 25/32.1-44.4/48.5 | (11) |
| AgNP | 21-51 | *Chrysosporium keratinophilum*  ( Fungi) | *An.stephensi,*  *Cx.quinquefaciatus Ae. Aegypti* | 1-3th/4^th^  1-3th/4^th^  1^st^or 2^nd^/3^rd^/4^th^ | 24  1  1 | 6-12/6  */_  6/*/2 | 10-12.9/12.6  */_  13.5/*/10 | (12) |
| AgNP | 20-50 | *Verticillium lecanii* ( Fungi) | *An.stephensi,*  *Cx.quinquefasciatus*  *Ae. aegypti* | 1^st^ 2nd/3^rd^/4^th^  1^st^or2^nd^/3^rd^/4^th^  1^st^ /2^nd^ /3^rd^or 4^th^ | 24  1 | */8/1.6/6  */2/_  4/_/* | */13.2/12/14.1  */11/_  12.3/_/* |
| AgNP | 15-55 | *Aquilaria sinensis* (Oil) | *Ae.albopictus* | 1-4^th^ /pupa | 2 | 0.8-1.1/0.9 | 1.7-4.4/2.1 | (13) |
| AgNP | 16-87 | *Pogostemon cablin* ( Oil) | *Ae. albopictus* | 1-4^th^ /pupa | 24 | 0.85-1.2/0.8 | 1.8-3.4/2.14 |
| AgNP | 16-80 | *Bacillus amyloliquefaciens* ( Bacteria) | *Cx.pipiens pallens* | 1^st^ -4^th^ /Pupa | 24 | 0.8-3.6/33.51 | 5.3-38.7/ 869.5 | (14) |
| AgNP | 16-80 | *Bacillus subtilis* ( Bacteria) | *Cx.pipiens pallens* | 1st-4th /Pupa | 24 | 0.34-1.9/13.8 | 4.2-26.5/124 |
| AgNP | 30-55 | *Aristolochia indica* ( Plant leaf) | *An. stephensi* | 1^st^-4^th^ /Pupa | 24 | 3.9- 10.5/15.7 | 17.4- 33.7/42 | (15) |
| PVP-AgNP | 65 | *Tridax procumbens* (Plant) | *An. stephensi* | 3^rd^or4^th^/Pupa | 48 | 1.6/1.7 | NA/NA | (16) |
| AgNP | 20--100 | *Annona* *reticulata* (Plant leaf) | *Ae. aegypti* | 4^th^ | 24 | 4.4 | 14 | (17) |
| AgNP | 25-40 | *Mimusops elengi* ( Plant leaf) | *An.stephensi*  *Ae. albopictus.* | 1^st^ -4^th^ /Pupa /Adult  1^st^-4^th^/Pupa/  Adult | 24  24 | 12.5-19.1/23.6/13.8  11.7- 17.8/21.5/14.8 | 31.3- 55.2/65.3/32.1  30.1- 51.9/62.5/33.9 | (18) |
| AgNP | 32 | *Bauhinia variegate (* Plant leaf) | *An.subpictus, Ae.albopictus,*  *Cx.tritaeniorhynchus* | 3^rd^  3^rd^  3^rd^ | 24  24  24 | 42  46.2  51.9 | 82.9  89.4  97.12 | (19) |
| AgNP | 32 | *Heliotropium indicum* (Pant leaf) | *An.stephensi,*  *Ae. aegypti,*  *Cx.quinquefasciatus* | Adult  Adult  Adult | 24  24  24 | 26.7  29.6  32.1 | 49.1  54.3  58.4 | (20) |
| AgNP | 30-37 | *Leucas aspera* ( Plant leaf) | *Ae. aegypti*  *An. stephensi* | 1^st^-4^th^ /Pupa  1st-4th /Pupa | 24  24 | 13.1-22.1/25.5  12.5-19.2/22.3 | 24.1-41.3/47.3  23.50-43/43 | (21) |
| AgNP | 50 | *Habenaria plantaginea* (Plant leaf) | *An. stephensi,*  *An.subpictus,*  *Ae. aegypti,*  *Cx.quinquefasciatus*  *Ae. albopictus,*  *Cx.tritaeniorhynchus* | 3^rd^  3^rd^  3^rd^  3^rd^  3^rd^  3^rd^ | 24  24  24  24  24  24 | 12.2  13.4  14.4  14.8  15.4  16.9 | 24  28.2  25.8  27.6  30.1  31.9 | (22) |
| AgNP | 43–79 | *Sargassum muticum*  ( seaweed) | *Ae. aegypti,*  *An. stephensi,*  *Cx quinquefasciatus* | 1^st^-4^th^ /Pupa  1-4^th^/Pupa  1-4^th^ /Pupa | 72  72  72 | 20.2- 28.1/30.6  16.2- 26.1/28.9  23.1- 30.2/32.6 | 46.6-65.3/71  42.4-62.2/67.6  52.4- 69.2/74.5 | (23) |
| AgNP | 5–35 | *Nicandra physalodes (* Plant leaf) | *An. stephensi,*  *Ae. aegypti*  *Cx.quinquefasciatus* | 3^rd^  3^rd^  3^rd^ | 24  24  24 | 12.4  13.6  14.8 | 24.2  25.7  27.3 | (24) |
| AgNP | 20 – 70 | *Aspergillus niger* ( Fungi) | *An.stephensi,*  *Cx.quinquefasciatus*  *Ae.aegypti.* | 1^st^-2^nd^/3^rd^ /4^th^ /Pupa  1-3th/4^th^/Pupa  1^st^/2^nd^/3^rd^/4^th^/pupa | 24  1/1/20  1/1/1/1/2 | */1.6/2/-  */-/6  5.6/-/*/4.7/4 | */8.9/1/_  _*/-/14_  _12.3/-/*/8/12_ | (25) |
| AgNP | 85 | *Delphinium denudatum* (Plant root) | *Ae. aegypti.* | 2^nd^ | 48 | 9.6 | NA | (26) |
| AgNP | 3- 31 | *Melia azedarach* ( Plant leaf) | *Ae.aegypti*  *Cx.quinquefasciatus* | 3^rd^  3^rd^ | 24  24 | 4.3  3.4 | 12.6  10.3 | (27) |
| AgNP | 20–35 | *Caulerpa Scalpelliformi* ( seaweed) | *Cx.quinquefasciatus.* | 1^st^ -4^th^/Pupa | 24 | 3.1- 5.9/7.3 | 7.5- 12.4/15.2 | (28) |
| AgNP | 41–60 | *Azadirachta indica* ( Plant leaf) | *Ae. aegypti*  *Cx.quinquefasciatus* | 3^rd^  3^rd^ | 24  24 | 0.006  0.05 | 0.04  0.23 | (29) |
| Ag NP | 25–59 | *Chrysanthemum indicum L* (Plant leaf) | *An. stephensi.* | 1^st-4th^ /Pupa | 24 | 5.1- 22.8/35.1 | 29.2- 88/115.1 | (30) |
| AgNP | 25–80 | *Leucas aspera* ( Plant leaf) | *Ae.aegypti* | 4^th^ | 24 | 8.6 | 21.6 | (31) |
| AgNP | 60–95 | *Morinda Tinctoria* (Plant leaf) | *Cx. quinquefasciatus* | 3^rd^ | 24 | 1.4 | NA | (32) |
| AgNP | 49 | *Beauveria bassiana* ( Fungi) | *Cx. Pipiens* | 2^nd^--4^th^ /Pupa | 24/48 | NA/250 | 150 /NA | (33) |
| AgNP | 30–60 | *Euphorbia hirta* ( Plant leaf*)* | *An.stephensi* | 1^st^ -4^th^ /pupa | 24 | 10.1- 27.9/34.5 | 32-69.9/79.8 | (34) |
| AgNP | 10.5- 19.2 | *Azadirachta indica* ( Plant bark) | *Cx.quinquefasciatus*  *An. stephensi* | 1^st^ -2^nd^ /3^rd^/4^th^ /Pupa /Adult  1^st^ -2^nd^ /3^rd^/4^th^ /pupa /adult | 0.5/1/1 /3/4  1/1/1/24/24 | */*/2/4/1.1  */_/_/_/_ | */*/12/11/2.1  */_/_/_/_ | (35) |
| AgNP | 15-19.2 | *Azadirachta indica* ( Plant leaf*)* | *Cx.quinquefasciatus*  *An. stephensi* | 1^st^/2^nd^/3^rd^/4^th^/Pupa/adult  1^st^/2^nd^ /3^rd^/4^th^/Pupa &Adult | 0.25/1.5/1.5/1.5/3/4  4/5/12/17/24 | */6/10/+/1/0.5  2/2/2/1/_ | */12/18/+/8/2.7  10/9/10/8/_ |
| AgNP | 29.6-62 | *Listeria monocytogenes (* Bacteria) | *Cx.quinquefasciatus*  *An. stephensi* | 1^st^ or 2^nd^/3^rd^or 4^th^ /Pupa/Adult  1^st^ or 2^nd^ /3^rd^ or 4^th^/Pupa /Adult | 0.25/0.75/5/5  72/72/24/2.5 | */*/*/_  */1/*/0.2 | */*/*/_  */8/*/3 | (36) |
| AgNP | 29.6-76.6 | *Bacillus subtilis* ( Bacteia) | *Cx. quinquefasciatus* | 1^st^ or 2^nd^ /3^rd^ /4^th^ /Pupa /Adult | 24/24/24/4/4 | */2/2.7/*/_ | */10.5/5.3/*/_ |
| AgNP | 11.5- 42.4 | *Streptomyces anulatus* ( Bacteria) | *Cx. quinquefasciatus*  *An. stephensi* | 1^st^/2^nd^/3^rd^/4^th^/Pupa /Adult  1st-4^th^ /Pupa /Adult | 0.25/3/8/1.5/4/4  5/2.5/2.5 | */*/*/*/*/_  */20/0.1 | */*/*/*/8/-  */120/1.3 |
| AgNP | 56–87 | *Cymodocea*  *serrulata* ( seagrass) | *Ae. aegypti,*  *Cx.quinquefasciatus*  *An.stephensi* | 1^st^-4^th^  1^st^ -4^th^  ^1st-4th^ | 24  24  24 | 4.4-9.2  7.4-13.2  2-5.5 | 67-361.5  302.8-434.4  36.4- 238.7 | (37) |
| AgNP | 24–51 | *Chrysosporium keratinophilum* ( Fungi) | *Cx. quinquefasciatus.* | Adult | 24 | 0.2 | 2.4 | (38) |
| AgNP | 20–50 | *Lecanicillium lecanii* ( Fungi) | *Cx. quinquefasciatus.* | Adult | 24 | 0.4 | 3.2 |
| AgNP | 20–40 | *Fusarium oxysorum* ( Fungus) | *Cx. quinquefasciatus.* | Adult | 24 | 0.4 | 4 |
| AgNP | 20-45 | *Agaricus bisporus* ( Bacteria) | *Cx quinquefasciatus* | 4^th^ | 18 | * | * | (39) |
| AgNP | 20-55 | *Escherichia coli* ( Bacteria) | *Cx quinquefasciatus* | 4^th^ | 24 | * | * |
| AgNP | 18-65 | *Penicillium* sp*.*( Bacteria) | *Cx quinquefasciatus* | 4^th^ | 24 | * | * |
| AgNP |  | *Vibrio* sp ( Bacteria) | *Cx quinquefasciatus* | 4^th^ | 24 | * | * |
| AgNP | ~35.42 | *Bacillus subtilis* ( Bacteria) | *Ae. aegypti*  *An. stephensi* | 4^th^  4^th^ | 24  24 | 4.3  4.2 | 14.1  12.9 | (40) |
| AgNP | 3.4-71.6 | *Andrographis serpyllifolia* (Plant leaf) | *Cx. Quinquefasciatu* | 3^rd^ | 24 | 68.9 | 392.1 | (41) |
| AgNP | 11.8 | *Cinnamomum zeylanicum* (Plant bark) | *An. stephensi*  *Cx. quinquefasciatus* | 1^st^/2^nd^/3^rd^/4^th^  1^st^/2^nd^/3^rd^/4^th^ | 4/4/22/22  24/24/24/24 | 2/10/6/1  */2/1.5/4 | 11/15/11/15  */11/10.5/13 | (42) |
| AgNP | 95 | *Lactobacillus* sp (Bacteria) | *Ae. aegypti* | 3^rd^ | 24 | 0.3 | 0.9 | (5) |
| AgNP | 137 | *Bacillus sp* | *Ae. aegypti* | 3^rd^ | 24 | 0.3 | 1.2 |
| AuNP | 46.5 | *Cinnamomum zeylanicum* (Plant leaf ) | *An.stephensi*  *Cx. quinquefasciatus* | 1^st^/2^nd^/3^rd^/4^th^  1^st^-4^th^ | 24/24/72/72  24 | */1/1/2  _ | */8/8/10  _ | (42) |
| Au–PdNP | 2-20 | *Citrus limon* (Plant leaf) | *An. stephensi*  *Ae. aegypti* | 1^st^-4^th^  1^st^/2^nd^/3^rd^/4^th^ | 24  24 | 5.1-11.4  12.4/11.2/6.2/10.8 | NA  NA/NA/NA/NA | (43) |
| AuNP | 50-100 | *Artemisia vulgaris L* (Plant leaf*).* | *Ae. aegypti L* | 3^rd^/3rd  4^th^ /4^th^ | 12/24  12/24 | 156.6/62.5  97. 9/43 | 2506.2/430.2  1677.4/376.7 | (44) |
| AuNP | 20-50 | *Chrysosporium keratinophilum* ( Fungi) | *An.stephensi,*  *Cx.quinquefasciatus*  *Ae. aegypti.* | 1^st^-4^th^  1^st^/2^nd^ -4^th^  1^st^/2^nd^/3^rd^/4^th^ | 72  72  72 | *  */12-30  12/12/24/30 | *  */36- 43.7  33.9/36.3/40.7/47.9 | (12) |
| AuNP | 1-40 | *Verticillium lecanii* ( Fungi) | *An.stephensi,*  *Cx quinquefasciatus Ae. aegypti* | 1^st^-4^th^  ^1st-4th^  ^1th -2nd /3rd/4th^ | 72  72  72/72/72 | _  *  */30/30 | _  *  */45.7/50.1 |
| AuNP | 20- 30 | *Entomophthra culicis*  (Fungi ) | *An.stphensi* | 1^st^/3^rd^/4^th^ /Pupa | 1/1/1/2 | */NA/4/8 | */8/8/NA | (45) |
| AuNP | 10–30 | *Aspergillus niger* ( Fungi) | *An.stephensi,*  *Cx. quinquefasciatus*  *Ae. aegypti* | 1^st^/2^nd^/3^rd^/4^th^  ^1st-4th^  ^1st-2nd /3rd/4th^ | 72  48  72 | */1.65/1.7/12  *  */24/30 | */24/30/39.8  *  8/37.2/45.70 | (46) |
| AuNP | 20 | *Anthocephalus cadamba* (Plant leaf) | *Cx. quinquefasciatus* | 1^st^ -4^th^ /Pupa | 24 | 0.7- 1.3/1.9 | 2.3- 3.2/4.2 | (47) |
| CuNP | 15.7–62.6 | *Metarhizium robertsii* ( Fungi) | *An.stephensi,*  *Ae.aegypti,*  *Cx quinquefasciatus* | 4^th^  4^th^  4^th^ | 24  24  24 | 3.5  7.8  3.1 | 23.1  65.1  15 | (48) |
| CuNP | 50–70 | *Tetramethyl orthosilica* ( Chemical) | *Cx. quinquefasciatus* | 1^st^/2^nd^/3^rd^/4^th^ | 24 | 157 .1/2028/ 890.1/3564.5 | NA/NA/NA/NA | (49) |
| CuNP | 16 | *Tridax procumbens (*  Plant leaf) | *Ae. aegypti* | 3^rd^ | 24 | 4.2 | NA | (50) |
| CuNP | 35–80 | *Extract copper* *(*Chemical) | *An. subpictus*  *Cx.quinquefasciatus* | 3^rd^  3^rd^ | 24  24 | 0.95  1.4 | NA  NA | (51) |
| SeNP |  | *Streptomyces* Sp *(*Actinobacterial ) | *Ae. aegypti, An.subpictus,*  *Cx. quinquefasciatus* | 4^th^  4^th^  4^th^ | 24  24  24 | 20  7.7  11.9 | 41.8  12.8  18.1 | (52) |
| SeNP | 46.3 -78.9 | *Clausena dentate* ( Plant leaf) | *An. stephensi,*  *An. aegypti,*  *Cx.quinquefasciatus* | 4^th^  4^th^  4^th^ | 24  24  24 | 240.7  104.1  99.6 | 367  158.1  153.6 | (53) |
| Ni NP | 34 | Solution purification ( Chemical) | *An. subpictus,*  *Cx. quinquefasciatus* | 4^th^  4^th^ | 24  24 | 6  6 | NA  NA | (54) |
| ZnO NP | 40-50 | *Sargassum wightii* ( seaweeds) | *Ae. aegypti* | 3^rd^ | 24 | 49.2 | 87 | (55) |
| ZnONP | 29.4-41.4 | *Lawsonia inermis* ( Plant) | *An. stephensi.* | 1^st^- 4^th^ /Pupa | 24 | 4.5-10.7/12.7 | NA/NA | (56) |
| ZnONP | 10–50 | *Ulva lactuca* ( Seaweed) | *Ae. aegypti* | 4^th^ | 24 | * | * | (57) |
| ZnONP | 100 | Powder *(* Chemical) | *Cx. quinquefasciatus* | 2^nd^ | 14day | 1 | 10 | (58) |
| ZnONP | <100nm | *Chemical* | *Ae.albopictus*  *An vagus larvae* | 2^nd^  2^nd^ | 24  24 | 68.4  13.6 | NA  NA | (59) |
| TiO_2_NP | 20–50 | *Parthenium hysterophorus* ( Plant leaf) | *Ae. aegypti*  *Cx.quinquefasciatus* | 4^th^  4^th^ | 24  24 | 11.6  8.7 | NA | (60) |
| Tio_2_ NP | 30±5 | *Mangifera indica* ( Plant leaf*)* | *An.subpictus, Cx.quinquefasciatus* | 4^th^  4^th^ | 24  24 | 7.7  8.1 | NA  NA | (61) |
| TiO_2_NP | 32- 37 | *Pleurotus sajor caju*  (Fruit) | *Ae. aegypti*  *Cx.quinquefasciatus* | 4^th^  4^th^ | 24  24 | 14.6  26.6 | 12.7  24.4 | (62) |
| TiO_2_ | 70.8 | Powder | *Ae. aegypti* | 1^st^-4^th/pupa^ | 24 | 4- 6.5/7.5 | 8.5-13.1/14.9 | (63) |
| MgO-NPs | 7–40 | *Penicillium chrysogenum* ( Fungi) | *An. stephensi* | 1^st^-4^th^ /Pupa | 24 | 12.4-15.5/16.5 | 22.3-28/29.8 | (64) |
| AL_2_O_3_ NP | 2.8-12 | *Aluminum acetate basic hydrate* ( Chemical) | *Cx.quinquefasciatus* | 2^nd^ | 24 | 11.1 | 35.42 | (65) |
| Co NPs | 84.8 | *Bacillus thuringiensis* ( Bacteria) | *An. subpictus*  *Ae. aegypti* | 4^th^  4^th^ | 24  24 | 3.6  2.87 | NA  NA | (66) |
| Fe NP | 35 -40 | *Ficus natalensis* (Plant leaf) | *Cx.quinquefasciatus* | 1st-4th /Pupa | 24 | 20.9-35.9/43.7 | 45.4 -81.2/93.4 | (67) |
| Fe_2_O_3_ | 8 | Chemical | *Cx.quinquefasciatus* | 1^st^-4^th^ /Pupa | 24 | 4.5 - 18.4 /22.1 | 15.2-40.8 /43 |
| PdNPs | 60 | *Tinospora cordifolia* ( Plant leaf) | *Cx.quinquefasciatus*  *An. subpictus.* | 1^st^-4^th^  1^st^-4^th^ | 24  24 | 3.3-6.1  2.9- 6.5 | 8.3- 13.7  6.0-13.8 | (68) |
| CdNP | 45-80 | *Tagetes* sp (flower). | *Ae.albopictus* | 4^th^ | 72 | * | * | (69) |
| CdNP | 45-60 | *Rosa* sp.(flower) | *Ae.albopictus* | 4^th^ | 72 | NA | 10 |

*=100 Mortality, _=No Mortality, PVP-AgNPs – polyvinyl silver nanoparticle, NA= not applicable.

**References**

1. Sujitha V, Murugan K, Paulpandi M, Panneerselvam C, Suresh U, Roni M, et al. Green-synthesized silver nanoparticles as a novel control tool against dengue virus (DEN-2) and its primary vector Aedes aegypti. Parasitology Research. 2015;114(9):3315-25.

2. El-Bendary MA, Moharam ME, Abdelraof M, Allam MA, Roshdy AM, Shaheen MN, et al. Multi-bioactive silver nanoparticles synthesized using mosquitocidal Bacilli and their characterization. Archives of Microbiology. 2020 Jan;202(1):63-75.

3. Morejón B, Pilaquinga F, Domenech F, Ganchala D, Debut A, Neira M. Larvicidal activity of silver nanoparticles synthesized using extracts of Ambrosia arborescens (Asteraceae) to control Aedes aegypti L.(Diptera: Culicidae). Journal of Nanotechnology. 2018;2018.

4. Singh G, Prakash S. Virulency of novel nanolarvicide from Trichoderma atroviride against Aedes aegypti (Linn.): a CLSM analysis. Environmental Science and Pollution Research. 2015;22(16):12559-65.

5. Wilson JJ, Deepalakshmi U, Ponmanickam P, Sivakumar T. Mosquito larvicidal activity of synthesized silver nanoparticles from selected bacteria against Aedes aegypti. Journal of Entomological Research. 2021;45(1):1-12.

6. Muthukumaran U, Govindarajan M, Rajeswary M. Green synthesis of silver nanoparticles from Cassia roxburghii—a most potent power for mosquito control. Parasitology Research. 2015;114(12):4385-95.

7. Chitra G, Balasubramani G, Ramkumar R, Sowmiya R, Perumal P. Mukia maderaspatana (Cucurbitaceae) extract-mediated synthesis of silver nanoparticles to control Culex quinquefasciatus and Aedes aegypti (Diptera: Culicidae). Parasitology Research. 2015;114(4):1407-15.

8. Muthukumaran U, Govindarajan M, Rajeswary M. Mosquito larvicidal potential of silver nanoparticles synthesized using Chomelia asiatica (Rubiaceae) against Anopheles stephensi, Aedes aegypti, and Culex quinquefasciatus (Diptera: Culicidae). Parasitology Research. 2015;114(3):989-99.

9. Pilaquinga F, Morejón B, Ganchala D, Morey J, Piña N, Debut A, et al. Green synthesis of silver nanoparticles using Solanum mammosum L.(Solanaceae) fruit extract and their larvicidal activity against Aedes aegypti L.(Diptera: Culicidae). PLoS One. 2019;14(10):e0224109.

10. Banu AN, Balasubramanian C. Myco-synthesis of silver nanoparticles using Beauveria bassiana against dengue vector, Aedes aegypti (Diptera: Culicidae). Parasitology research. 2014;113(8):2869-77.

11. Rajaganesh R, Murugan K, Panneerselvam C, Jayashanthini S, Roni M, Suresh U, et al. Fern-synthesized silver nanocrystals: towards a new class of mosquito oviposition deterrents? Research in Veterinary Science. 2016;109:40-51.

12. Soni N, Prakash S. Microbial synthesis of spherical nanosilver and nanogold for mosquito control. Annals of Microbiology. 2014;64(3):1099-111.

13. Ga’al H, Fouad H, Mao G, Tian J, Jianchu M. Larvicidal and pupicidal evaluation of silver nanoparticles synthesized using Aquilaria sinensis and Pogostemon cablin essential oils against dengue and zika viruses vector Aedes albopictus mosquito and its histopathological analysis. Artificial cells, Nanomedicine, and Biotechnology. 2018;46(6):1171-9.

14. Fouad H, Hongjie L, Yanmei D, Baoting Y, El-Shakh A, Abbas G, et al. Synthesis and characterization of silver nanoparticles using Bacillus amyloliquefaciens and Bacillus subtilis to control filarial vector Culex pipiens pallens and its antimicrobial activity. Artificial Cells, Nanomedicine, and Biotechnology. 2017;45(7):1369-78.

15. Murugan K, Labeeba MA, Panneerselvam C, Dinesh D, Suresh U, Subramaniam J, et al. Aristolochia indica green-synthesized silver nanoparticles: a sustainable control tool against the malaria vector Anopheles stephensi? Research in Veterinary Science. 2015;102:127-35.

16. Nyakundi EO, Padmanabhan MN. Green chemistry focus on optimization of silver nanoparticles using response surface methodology (RSM) and mosquitocidal activity: Anopheles stephensi (Diptera: Culicidae). Spectrochimica Acta Part A: Molecular and Biomolecular Spectroscopy. 2015;149:978-84.

17. Parthiban E, Manivannan N, Ramanibai R, Mathivanan N. Green synthesis of silver-nanoparticles from Annona reticulata leaves aqueous extract and its mosquito larvicidal and anti-microbial activity on human pathogens. Biotechnology Reports. 2019;21:e00297.

18. Subramaniam J, Murugan K, Panneerselvam C, Kovendan K, Madhiyazhagan P, Kumar PM, et al. Eco-friendly control of malaria and arbovirus vectors using the mosquitofish Gambusia affinis and ultra-low dosages of Mimusops elengi-synthesized silver nanoparticles: towards an integrative approach? Environmental Science and Pollution Research. 2015;22(24):20067-83.

19. Govindarajan M, Rajeswary M, Veerakumar K, Muthukumaran U, Hoti S, Mehlhorn H, et al: Novel synthesis of silver nanoparticles using Bauhinia variegata: a recent eco-friendly approach for mosquito control. Parasitology Research. 2016;115(2):723-33.

20. Veerakumar K, Govindarajan M, Hoti S. Evaluation of plant-mediated synthesized silver nanoparticles against vector mosquitoes. Parasitology Research. 2014;113(12):4567-77.

21. Sivapriyajothi S, Kumar PM, Kovendan K, Subramaniam J, Murugan K. Larvicidal and pupicidal activity of synthesized silver nanoparticles using Leucas aspera leaf extract against mosquito vectors, Aedes aegypti and Anopheles stephensi. Journal of Entomological and Acarological Research. 2014;46(2):77-84.

22. Aarthi C, Govindarajan M, Rajaraman P, Alharbi NS, Kadaikunnan S, Khaled JM, et al. Eco-friendly and cost-effective Ag nanocrystals fabricated using the leaf extract of Habenaria plantaginea: toxicity on six mosquito vectors and four non-target species. Environmental Science and Pollution Research. 2018;25(11):10317-27.

23. Madhiyazhagan P, Murugan K, Kumar AN, Nataraj T, Dinesh D, Panneerselvam C, et al. S argassum muticum-synthesized silver nanoparticles: An effective control tool against mosquito vectors and bacterial pathogens. Parasitology Research. 2015;114(11):4305-17.

24. Govindarajan M, Khater HF, Panneerselvam C, Benelli G. One-pot fabrication of silver nanocrystals using Nicandra physalodes: A novel route for mosquito vector control with moderate toxicity on non-target water bugs. Research in Veterinary Science. 2016;107:95-101.

25. Soni N, Prakash S. Possible mosquito control by silver nanoparticles synthesized by soil fungus (Aspergillus niger 2587). Advances in Nanoparticles. 2013;2:125-32.

26. Suresh G, Gunasekar PH, Kokila D, Prabhu D, Dinesh D, Ravichandran N, et al. Green synthesis of silver nanoparticles using Delphinium denudatum root extract exhibits antibacterial and mosquito larvicidal activities. Spectrochimica Acta Part A: Molecular and Biomolecular Spectroscopy. 2014;127:61-6.

27. Ramanibai R, Velayutham K. Bioactive compound synthesis of Ag nanoparticles from leaves of Melia azedarach and its control for mosquito larvae. Research in Veterinary Science. 2015;98:82-8.

28. Murugan K, Benelli G, Ayyappan S, Dinesh D, Panneerselvam C, Nicoletti M, et al. Toxicity of seaweed-synthesized silver nanoparticles against the filariasis vector Culex quinquefasciatus and its impact on predation efficiency of the cyclopoid crustacean Mesocyclops longisetus. Parasitology Research. 2015;114(6):2243-53.

29. Poopathi S, De Britto LJ, Praba VL, Mani C, Praveen M. Synthesis of silver nanoparticles from Azadirachta indica—a most effective method for mosquito control. Environmental Science and Pollution Research. 2015;22(4):2956-63.

30. Arokiyaraj S, Dinesh Kumar V, Elakya V, Kamala T, Park SK, Ragam M, et al. Biosynthesized silver nanoparticles using floral extract of Chrysanthemum indicum L.—potential for malaria vector control. Environmental Science and Pollution Research. 2015;22(13):9759-65.

31. Suganya G, Karthi S, Shivakumar MS. Larvicidal potential of silver nanoparticles synthesized from Leucas aspera leaf extracts against dengue vector Aedes aegypti. Parasitology Research. 2014;113(3):875-80.

32. Kumar KR, Nattuthurai N, Gopinath P, Mariappan T. Synthesis of eco-friendly silver nanoparticles from Morinda tinctoria leaf extract and its larvicidal activity against Culex quinquefasciatus. Parasitology Research. 2015;114(2):411-7.

33. Qamandar MA, Shafeeq MAA. Possible Mosquito Control by Silver Nanoparticles Synthesized by Entomopathogenic Fungus Beauveria bassiana. Research Journal of Pharmacy and Technology. 2018;11(3):1058-64.

34. Agalya Priyadarshini K, Murugan K, Panneerselvam C, Ponarulselvam S, Hwang J-S, Nicoletti M. Biolarvicidal and pupicidal potential of silver nanoparticles synthesized using Euphorbia hirta against Anopheles stephensi Liston (Diptera: Culicidae). Parasitology Research. 2012;111(3):997-1006.

35. Soni N, Prakash S. Silver nanoparticles: a possibility for malarial and filarial vector control technology. Parasitology Research. 2014;113(11):4015-22.

36. Soni N, Prakash S. Antimicrobial and mosquitocidal activity of microbial synthesized silver nanoparticles. Parasitology Research. 2015;114(3):1023-30.

37. Amutha V, Deepak P, Kamaraj C, Balasubramani G, Aiswarya D, Arul D, et al. Mosquito-larvicidal potential of metal and oxide nanoparticles synthesized from aqueous extract of the seagrass, Cymodocea serrulata. Journal of Cluster Science. 2019;30(3):797-812.

38. Soni N, Prakash S. Fungal-mediated nano silver: an effective adulticide against mosquito. Parasitology Research. 2012;111(5):2091-8.

39. Dhanasekaran D, Thangaraj R. Evaluation of larvicidal activity of biogenic nanoparticles against filariasis causing Culex mosquito vector. Asian Pacific Journal of Tropical Disease. 2013;3(3):174-9.

40. Nanda A, Lakshmipathy M, Elumalai D, Kaleena P, Nayak B. Application of biosynthesized silver nanoparticles as a novel vector control agent. Der Pharmacutical Letters. 2015;7(8):228-31.

41. Madhankumar R, Sivasankar P, Kalaimurugan D, Murugesan S. Antibacterial and larvicidal activity of silver nanoparticles synthesized by the leaf extract of Andrographis serpyllifolia wight. Journal of Cluster Science. 2020;31(4):719-26.

42. Soni N, Prakash S. Green nanoparticles for mosquito control. The Scientific World Journal. 2014;2014.

43. Minal SP, Prakash S. Laboratory analysis of Au–Pd bimetallic nanoparticles synthesized with Citrus limon leaf extract and its efficacy on mosquito larvae and non-target organisms. Scientific Reports. 2020;10(1):1-13.

44. Sundararajan B, Kumari BR. Novel synthesis of gold nanoparticles using Artemisia vulgaris L. leaf extract and their efficacy of larvicidal activity against dengue fever vector Aedes aegypti L. Journal of Trace Elements in Medicine and Biology. 2017;43:187-96.

45. H.R. M. Toxicity of golden nanoparticles synthesized by Insects pathogen Entomophthra culicis versus mosquito Anopheles stephensi (Diptera:Culicidae) Advance Research Journal of Multidisciplinary Discoveries. 2016;8.0(7):44-8.

46. Soni N, Prakash S. Synthesis of gold nanoparticles by the fungus Aspergillus niger and its efficacy against mosquito larvae. Rep Parasitology. 2012;2(1).

47. Jeyalalitha T, Murugan K, Madhiyazhagan P. Bioefficacy of plant-mediated gold nanoparticles and Anthocepholus cadamba on filarial vector, Culex quinquefasciatus (Insecta: Diptera: Culicidae). Parasitology Research. 2013;112(3):1053-63.

48. Vivekanandhan P, Swathy K, Thomas A, Kweka EJ, Rahman A, Pittarate S, et al. Insecticidal efficacy of microbial-mediated synthesized copper nano-pesticide against insect pests and non-target organisms. International Journal of Environmental Research and Public Health. 2021;18(19):10536.

49. Chakrabarti A, Patra P. Relative larvicidal property of common oxide nanostructures against Culex quinquefasciatus. IET Nanobiotechnology. 2020;14(5):389-95.

50. Muthamil Selvan S, Vijai Anand K, Govindaraju K, Tamilselvan S, Kumar VG, Subramanian KS, et al. Green synthesis of copper oxide nanoparticles and mosquito larvicidal activity against dengue, zika and chikungunya causing vector Aedes aegypti. IET Nanobiotechnology. 2018;12(8):1042-6.

51. Ramyadevi J, Jeyasubramanian K, Marikani A, Rajakumar G, Rahuman AA, Santhoshkumar T, et al. Copper nanoparticles synthesized by polyol process used to control hematophagous parasites. Parasitology Research. 2011;109(5):1403-15.

52. Ramya S, Shanmugasundaram T, Balagurunathan R. Actinobacterial enzyme mediated synthesis of selenium nanoparticles for antibacterial, mosquito larvicidal and anthelminthic applications. Particulate Science and Technology. 2020;38(1):63-72.

53. Sowndarya P, Ramkumar G, Shivakumar M. Green synthesis of selenium nanoparticles conjugated Clausena dentata plant leaf extract and their insecticidal potential against mosquito vectors. Artificial Cells, Nanomedicine, and Biotechnology. 2017;45(8):1490-5.

54. Rajakumar G, Rahuman AA, Velayutham K, Ramyadevi J, Jeyasubramanian K, Marikani A, et al. Novel and simple approach using synthesized nickel nanoparticles to control blood-sucking parasites. Veterinary Parasitology. 2013;191(3-4):332-9.

55. Ishwarya R, Vaseeharan B, Subbaiah S, Nazar AK, Govindarajan M, Alharbi NS, et al. Sargassum wightii-synthesized ZnO nanoparticles–from antibacterial and insecticidal activity to immunostimulatory effects on the green tiger shrimp Penaeus semisulcatus. Journal of Photochemistry and Photobiology 2018;183:318-30.

56. Amuthavalli P, Hwang J-S, Dahms H-U, Wang L, Anitha J, Vasanthakumaran M, et al. Zinc oxide nanoparticles using plant Lawsonia inermis and their mosquitocidal, antimicrobial, anticancer applications showing moderate side effects. Scientific Reports. 2021;11(1):1-13.

57. Ishwarya R, Vaseeharan B, Kalyani S, Banumathi B, Govindarajan M, Alharbi NS, et al. Facile green synthesis of zinc oxide nanoparticles using Ulva lactuca seaweed extract and evaluation of their photocatalytic, antibiofilm and insecticidal activity. Journal of Photochemistry and Photobiology 2018;178:249-58.

58. Mostafa WA, Elgazzar E, Beall GW, Rashed SS, Rashad EM. Insecticidal effect of zinc oxide and aluminum oxide nanoparticles synthesized by co-precipitation technique on Culex quinquefasciatus larvae (Diptera: Culicidae). International Journal of Applied Research. 2018;4(4):290-7.

59. Gunathilaka U, de Silva W, Dunuweera S, Rajapakse R. Effect of morphology on larvicidal activity of chemically synthesized zinc oxide nanoparticles against mosquito vectors. RSC Advances. 2021;11(15):8857-66.

60. Thandapani K, Kathiravan M, Namasivayam E, Padiksan IA, Natesan G, Tiwari M, et al. Enhanced larvicidal, antibacterial, and photocatalytic efficacy of TiO2 nanohybrids green synthesized using the aqueous leaf extract of Parthenium hysterophorus. Environmental Science and Pollution Research. 2018;25(11):10328-39.

61. Rajakumar G, Rahuman AA, Roopan SM, Chung I-M, Anbarasan K, Karthikeyan V. Efficacy of larvicidal activity of green synthesized titanium dioxide nanoparticles using Mangifera indica extract against blood-feeding parasites. Parasitology Research. 2015;114(2):571-81.

62. Manimaran K, Natarajan D, Balasubramani G, Murugesan S. Pleurotus sajor caju Mediated TiO2 Nanoparticles: A Novel Source for Control of Mosquito Larvae, Human Pathogenic Bacteria and Bone Cancer Cells. Journal of Cluster Science. 2022;33(4):1489-99.

63. Murugan K, Dinesh D, Kavithaa K, Paulpandi M, Ponraj T, Alsalhi MS, et al. Hydrothermal synthesis of titanium dioxide nanoparticles: mosquitocidal potential and anticancer activity on human breast cancer cells (MCF-7). Parasitology Research. 2016;115(3):1085-96.

64. Fouda A, Awad MA, Eid AM, Saied E, Barghoth MG, Hamza MF, et al. An eco-friendly approach to the control of pathogenic microbes and Anopheles stephensi malarial vector using magnesium oxide nanoparticles (Mg-nps) fabricated by Penicillium chrysogenum. International Journal of Molecular Sciences. 2021;22(10):5096.

65. Mostafa WA, Elgazzar E, Beall GW, Rashed SS, Rashad EM. Insecticidal effect of zinc oxide and aluminum oxide nanoparticles synthesized by co-precipitation technique on Culex quinquefasciatus larvae (Diptera: Culicidae). International Journal of Applied Research. 2018;4(4):290-7.

66. Marimuthu S, Rahuman AA, Kirthi AV, Santhoshkumar T, Jayaseelan C, Rajakumar G. Eco-friendly microbial route to synthesize cobalt nanoparticles using Bacillus thuringiensis against malaria and dengue vectors. Parasitology Research. 2013;112(12):4105-12.

67. Murugan K, Dinesh D, Nataraj D, Subramaniam J, Amuthavalli P, Madhavan J, et al. Iron and iron oxide nanoparticles are highly toxic to Culex quinquefasciatus with little non-target effects on larvivorous fishes. Environmental Science and Pollution Research. 2018;25(11):10504-14.

68. Jayaseelan C, Gandhi PR, Rajasree SRR, Suman TY, Mary RR. Toxicity studies of nanofabricated palladium against filariasis and malaria vectors. Environmental Science and Pollution Research. 2018;25(1):324-32.

69. Hajra A, Dutta S, Mondal NK. Mosquito larvicidal activity of cadmium nanoparticles synthesized from petal extracts of marigold (Tagetes sp.) and rose (Rosa sp.) flower. Journal of Parasitic Diseases. 2016;40(4):1519-27.
